# Evaluating Artificial Intelligence Models in Dermatology: Comparative Analysis

**DOI:** 10.2196/74040

**Published:** 2025-12-04

**Authors:** Aneri Bhargav Patel, William Driscoll, Conan H Lee, Cameron Zachary, Nicole M Golbari, Janellen Smith

**Affiliations:** 1 Department of Dermatology, University of California, Irvine, 850 Health Sciences Road, 2nd floor, Irvine, CA, 92617, United States, 1 9498240606; 2 School of Medicine, University of California, Davis, Sacramento, CA, United States; 3 New York Genomics, New York, NY, United States; 4 Department of Dermatology, University of California, Irvine, 850 Health Sciences Road, 2nd floor, Irvine, CA, 92617, United States; 5 Department of Dermatology, NYU Langone Health, New York, NY, United States

**Keywords:** ChatGPT, DermGPT, artificial intelligence, dermatology, education, LLM, large language model

## Abstract

DermGPT demonstrated strong potential for improving answer clarity and conciseness in dermatology-related queries, while ChatGPT provided more robust source citations, enhancing trust in evidence-based responses.

## Introduction

Large language models (LLMs) like OpenAI’s GPT-4o use transformer architectures with self-attention to process and generate human-like responses. ChatGPT, developed by OpenAI [1], enhances a GPT-4 model with reinforcement learning from human feedback, filtering inappropriate content [2]. These models predict the next word based on prior context. Trained on vast internet data, they can address diverse topics, including dermatology. However, LLMs may “hallucinate,” producing plausible but incorrect information [3,4], limiting clinical utility.

DermGPT [5], developed under the Palo Alto Medical Foundation, is tailored for dermatology. Beyond drafting notes and authorizations, it answers dermatology questions using a GPT base model enhanced by a research database. By sourcing answers from this material and showing citations, DermGPT aims to reduce hallucinations and better support dermatologists [6]. We compared its responses to those of ChatGPT.

## Methods

### Overview

ChatGPT was selected for its popularity and prior evidence of superiority in dermatology-related tasks. A double-blind study found dermatologists preferred ChatGPT over Google’s Bard for patient handouts [7]. ChatGPT 4o was used. DermGPT’s only available model was used.

Two dermatology residents, CZ and NMG, authored a list of questions posed to each LLM (Multimedia Appendix 1). Three questions to which DermGPT did not provide a response were excluded as nonevaluable item pairs. The two models’ answers for a given question were paired and assigned as A or B using a computer-generated randomization list. Any identifiable metadata such as formatting was cleared. The survey was distributed to dermatologists at the University of California, Irvine, and the University of California, Davis, via email and QR codes. Survey takers were informed that both responses were produced by LLMs, but they were blinded to which model produced which response. They were asked to choose their preferred answers based on quality—specifically, which answer they thought would be best suited for patient care or was most accurate.

The rating options were as follows:

- Model A better
- Model B better
- Equal quality
- Both inadequate

Statistical analysis was conducted using SAS OnDemand for Academics (version 9.4). *χ*^2^ tests (*P*<.05) assessed significance. Interrater reliability was not prespecified and not assessed; ratings were aggregated at the item level.

### Ethical Considerations

This study used a voluntary, anonymous survey of physicians and residents. According to institutional and national guidelines, the project did not require institutional review board review because no identifiable information was collected and the study posed minimal risk.

Participants provided implied consent by completing the survey after being informed of its purpose and their ability to withdraw at any time. No compensation was provided. The survey responses were analyzed in aggregate to ensure anonymity and privacy in accordance with institutional standards. The study followed the ethical principles of the Declaration of Helsinki, adhered to Committee on Publication Ethics guidelines**,** and met all institutional requirements for minimal-risk survey research.

## Results

### Overview

Of 64 dermatology faculty and 30 residents across the University of California, Irvine, and the University of California, Davis, we received a total of 19 responses, comprising 13 attending physicians and 6 residents or fellows. This corresponds to an overall response rate of approximately 20%.

### Which LLM’s Answer Was Better: ChatGPT or DermGPT?

Overall, DermGPT’s answers (48.1%) were preferred over ChatGPT’s (28.4%); the *χ*^2^ test was significant with *P*=.04 (*P*<.05). In the attending group, DermGPT’s answers were preferred (93/195, 47.7%) over ChatGPT’s (56/195, 28.7%). Likewise, in the resident group, DermGPT’s answers were preferred (44/90, 48.9%) versus ChatGPT (25/90, 27.8%) (Table 1).

**Table 1.** User-preferred artificial intelligence answer.

Group/responses	ChatGPT		DermGPT		Other		Total answers	
	Values, n (%)	Percentage of total responses	Values, n (%)	Percentage of total responses	Values, n (%)	Percentage of total responses	Responses	Percentage of total responses
Attending	56 (28.7)	19.6	93 (47.7)	32	46 (23.6)	16.1	195	68.4
Resident	25 (27.8)	8.8	44 (48.9)	15.4	21 (23.3)	7.4	90	31.6
Total	81	28.4	137	48.1	67	23.5	285	100

a *χ*2 test: *P*=.04.

### Which LLM’s References Were Better: ChatGPT or DermGPT?

Overall, ChatGPT references (46%) were preferred over DermGPT (23.5%; *χ*^2^_2_=1.385; *P*=.50). In the attending group, ChatGPT references were also preferred (94/195, 48.2%) over DermGPT (45/195, 23.1%). Likewise, in the resident group, ChatGPT references were preferred (37/90, 41.1%) versus DermGPT (22/90, 24.4%) (Table 2).

**Table 2.** Overall preference for references.^a^

Group	ChatGPT		DermGPT		Other		Total answers	
	Values, n (%)	Percent of total responses	Values, n (%)	Percent of total responses	Values, n (%)	Percent of total responses	Responses	Percent of total responses
Attending	94 (48.2)	33	45 (23.1)	15.8	56 (28.7)	19.6	195	68.4
Resident	37 (41.1)	13	22 (24.4)	7.7	31 (34.4)	10.9	90	31.6
Total	131	46	67	23.5	87	30.5	285	100

a *χ*2_2_=1.385; *P*=.50.

## Discussion

### Principal Results

Out of 195 responses, users generally preferred DermGPT’s answers, while ChatGPT was favored for its reference citations (Table 2). DermGPT’s concise and well-phrased responses made it accessible for quick clinical reference. However, 3 questions were excluded because DermGPT issued disclaimers instead of direct answers, recommending consultation with a dermatologist or guidelines. The multimedia appendices show the results tabulated from SAS as well as the questionnaire and responses (Multimedia Appendices 1-6).

ChatGPT consistently cited reputable references such as the *Journal of the American Academy of Dermatology* and the *Journal of the American Medical Association*, contributing to user trust and perceived academic rigor. Although DermGPT offers clarity, ChatGPT’s strong sourcing enhances credibility. These results suggest the potential for a hybrid model that combines both strengths.

### Limitations

Our study was constrained by a small rater sample (n=19) and multiple ratings per rater and per question. As a result, *P* values should be interpreted as exploratory rather than confirmatory. The sample may not represent all dermatology clinicians, limiting generalizability. Subgroup patterns were underpowered.

### Comparison With Prior Work

Several studies have compared LLMs to each other and to humans. He et al [8] found GPT-4 sometimes produced inaccurate, nonindividualized responses to laboratory-related queries. Iannantuono et al [9] compared ChatGPT-4, ChatGPT-3.5, and Google Bard in immunooncology, stressing the need for expert verification. Fernández-Pichel et al [10] found LLMs answered 80% of health questions accurately, though results were sensitive to prompt phrasing. This is the first study comparing ChatGPT and DermGPT for dermatologic responses.

### Conclusions and Future Directions

Future research should include models like Claude and Gemini, expand sample size, and explore combining DermGPT’s brevity with ChatGPT’s sourcing. These results highlight the importance of balancing clarity and citation in artificial intelligence–assisted medical tools.

## Supplementary material

10.2196/74040 Multimedia Appendix 1 Study process.

10.2196/74040 Multimedia Appendix 2 Survey questionnaire.

10.2196/74040 Multimedia Appendix 3 SAS results, part 1.

10.2196/74040 Multimedia Appendix 4 SAS results, part 2.

10.2196/74040 Multimedia Appendix 5 SAS results, part 3.

10.2196/74040 Multimedia Appendix 6 Survey questions and answers.

10.2196/74040 Multimedia Appendix 7 Comments from survey takers.

## Abbreviations

LLM: large language model
