# Supplementary figures and images for: Evaluating Artificial Intelligence Models in Dermatology: Comparative Analysis
Source: JMIR Dermatol. 2025 Dec 4;8:e74040. doi: 10.2196/74040 (PMC12677980; doi:10.2196/74040)

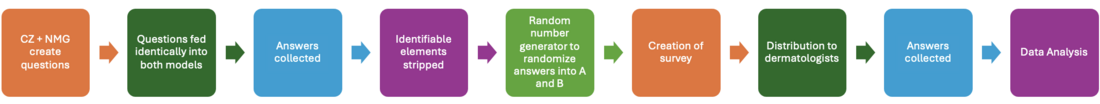

Supplement: Multimedia Appendix 1 [file derma-v8-e74040-s001.png]
