# Supplementary material for: Evaluating Artificial Intelligence Models in Dermatology: Comparative Analysis
Source: JMIR Dermatol. 2025 Dec 4;8:e74040. doi: 10.2196/74040 (PMC12677980; doi:10.2196/74040)
Supplement: Multimedia Appendix 2 [file derma-v8-e74040-s002.docx]

Chat GPT vs. Derm GPT

Hello, thank you for participating in this study! This is a 30 question survey, it will take approximately 15-20 minutes to finish. One of the answers is generated by Chat GPT and the other by Derm GPT.

Out of the two responses shown below, please pick the answer that best answers the question posed. If you think both answers are inadequate, you can pick that option below. **The sources for answers used by each model are listed as a second question, with source "A" corresponding to answer "A".** Please pick the source that is a better in terms of quality.

*For any questions/comments, please email: aneribp@hs.uci.edu*

* Indicates required question

1. Name *
2. Email *
3. Training level *

*Mark only one oval.*

Resident

Fellow

Attending

# What is the best treatment for mild psoriasis? *


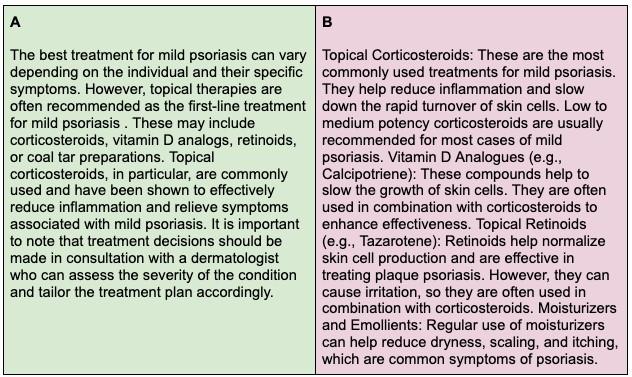


*Check all that apply.*

A is a better response

B is a better response

A and B are about equal in quality of response

I think both answers are inadequate in answering the posed question


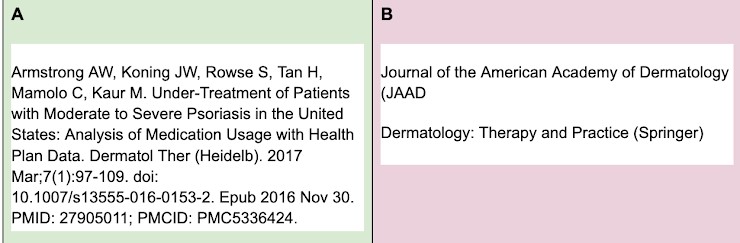


*Check all that apply.*

A has a better reference

B has a better reference

References in A and B are about equal in quality of response

I think both answers are inadequate in answering the posed question

# What is the difference between a papule and a macule? *


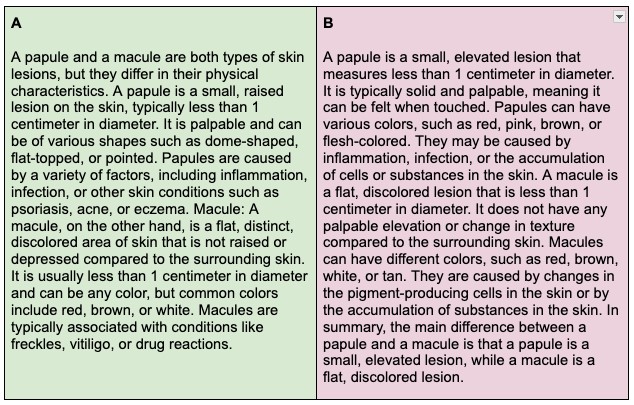


*Check all that apply.*

A is a better response

B is a better response

A and B are about equal in quality of response

I think both answers are inadequate in answering the posed question


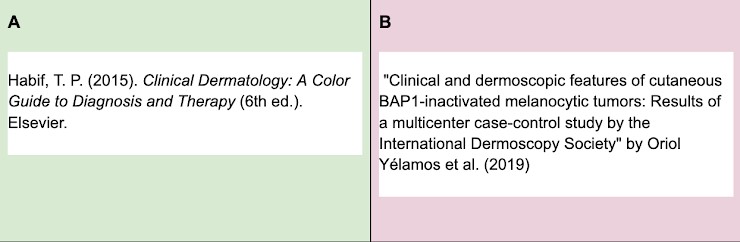


*Check all that apply.*

A has a better reference

B has a better reference

References in A and B are about equal in quality of response

I think both answers are inadequate in answering the posed question

# What are the most reported side effects of IL-23 inhibitors? *


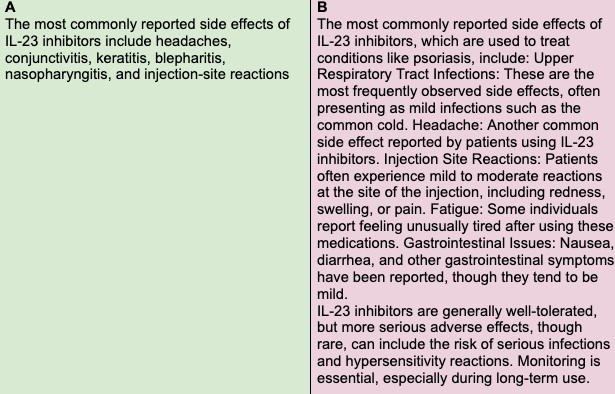


*Check all that apply.*

A is a better response

B is a better response

A and B are about equal in quality of response

I think both answers are inadequate in answering the posed question


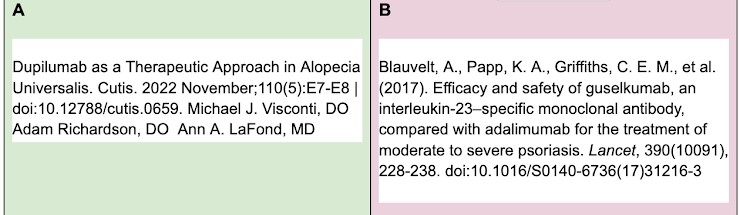


*Check all that apply.*

A has a better reference

B has a better reference

References in A and B are about equal in quality of response

I think both answers are inadequate in answering the posed question

# Describe the histological differences between eczema and psoriasis. *


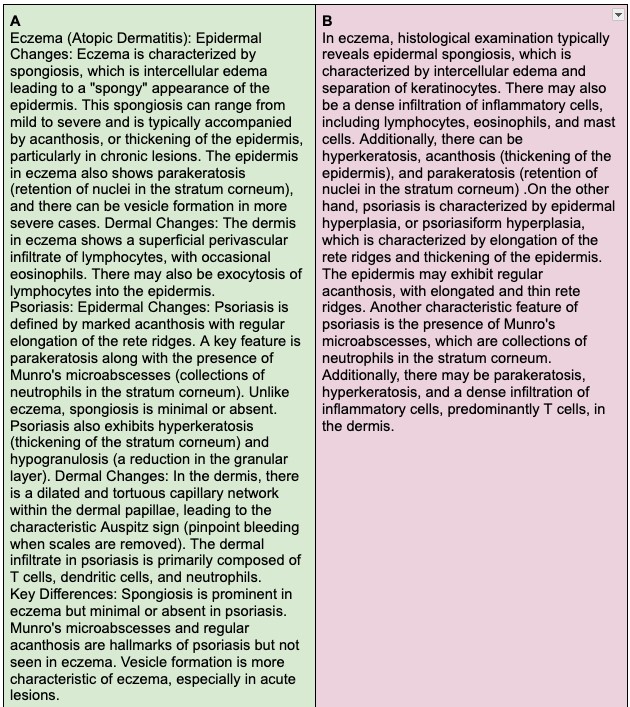


*Check all that apply.*

A is a better response

B is a better response

A and B are about equal in quality of response

I think both answers are inadequate in answering the posed question

Each source below aligns with answer above. Pick the better source in terms of quality.


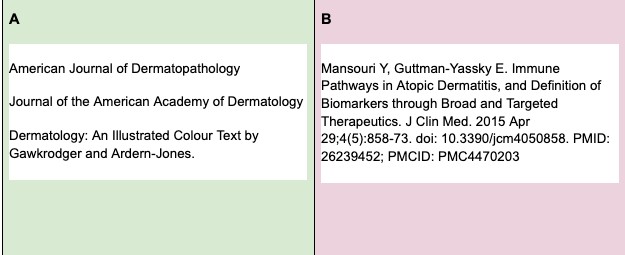


*Check all that apply.*

A has a better reference

B has a better reference

References in A and B are about equal in quality of response

I think both answers are inadequate in answering the posed question

# What is the etiology of Granuloma Annulare? *


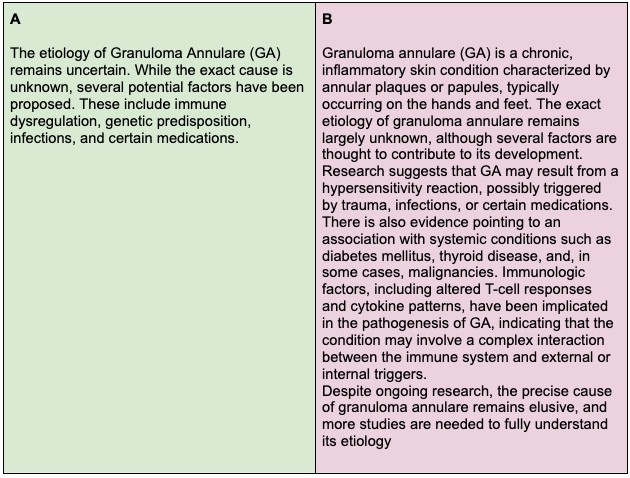


*Check all that apply.*

A is a better response

B is a better response

A and B are about equal in quality of response

I think both answers are inadequate in answering the posed question

Each source below aligns with answer above. Pick the better source in terms of quality.


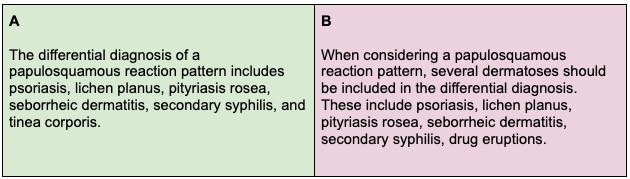


*Check all that apply.*

A is a better response

B is a better response

A and B are about equal in quality of response

I think both answers are inadequate in answering the posed question


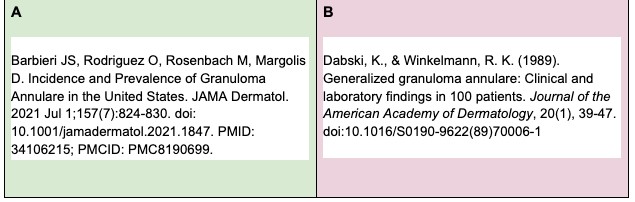


*Check all that apply.*

A has a better reference

B has a better reference

References in A and B are about equal in quality of response

I think both answers are inadequate in answering the posed question

14. **What dermatoses should be included in the differential diagnosis of a** * **papulosquamous reaction pattern?**


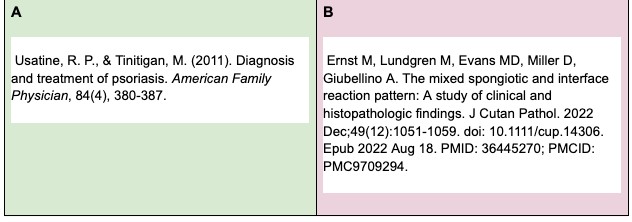


*Check all that apply.*

A has a better reference

B has a better reference

References in A and B are about equal in quality of response

I think both answers are inadequate in answering the posed question

**Bullous Pemphigoid is caused by an autoantibody response directed against which antigens?**


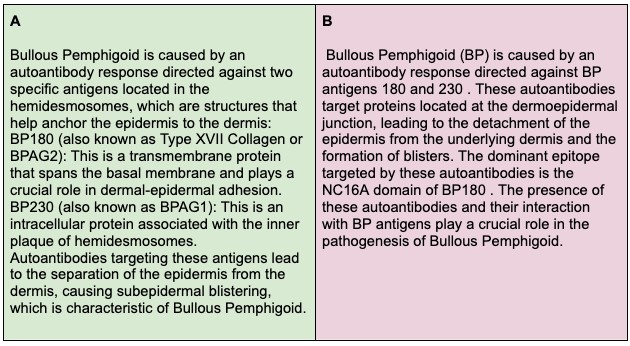


*Check all that apply.*

A is a better response

B is a better response

A and B are about equal in quality of response

I think both answers are inadequate in answering the posed question


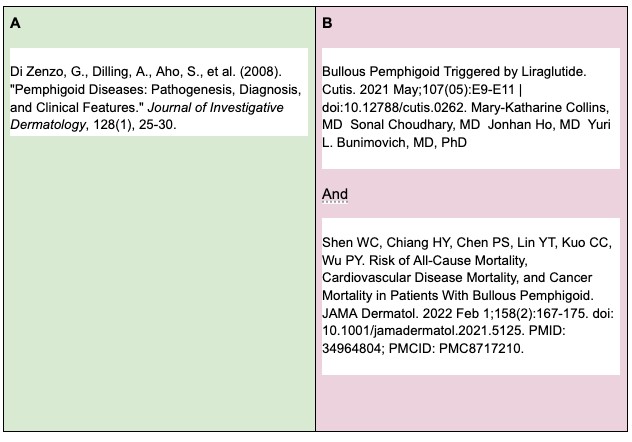


*Check all that apply.*

A has a better reference

B has a better reference

References in A and B are about equal in quality of response

I think both answers are inadequate in answering the posed question

# What are the guidelines for prescribing isotretinoin? *


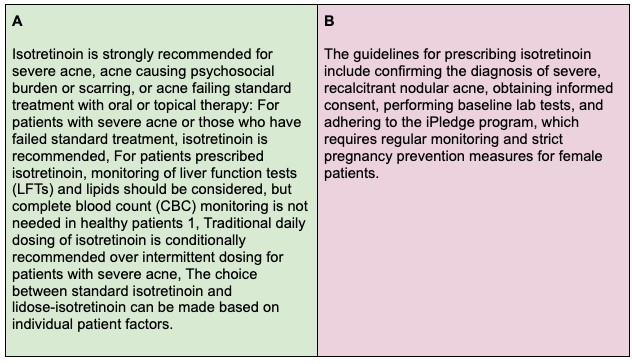


*Check all that apply.*

A is a better response

B is a better response

A and B are about equal in quality of response

I think both answers are inadequate in answering the posed question


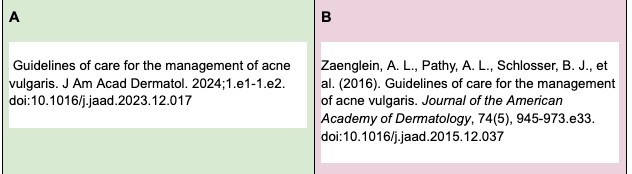


*Check all that apply.*

A has a better reference

B has a better reference

References in A and B are about equal in quality of response

I think both answers are inadequate in answering the posed question

# What is the classic clinical appearance of calciphylaxis? *


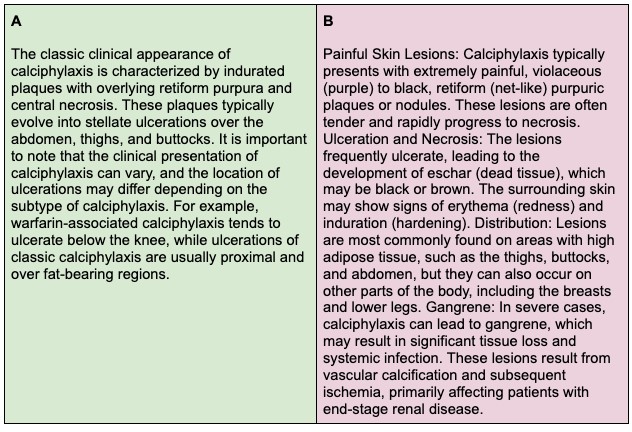


*Check all that apply.*

A is a better response

B is a better response

A and B are about equal in quality of response

I think both answers are inadequate in answering the posed question


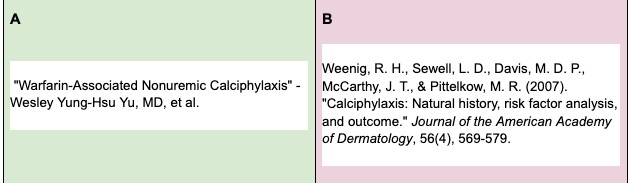


*Check all that apply.*

A has a better reference

B has a better reference

References in A and B are about equal in quality of response

I think both answers are inadequate in answering the posed question

# How do you rate the severity of Hidradenitis Suppurativa? *


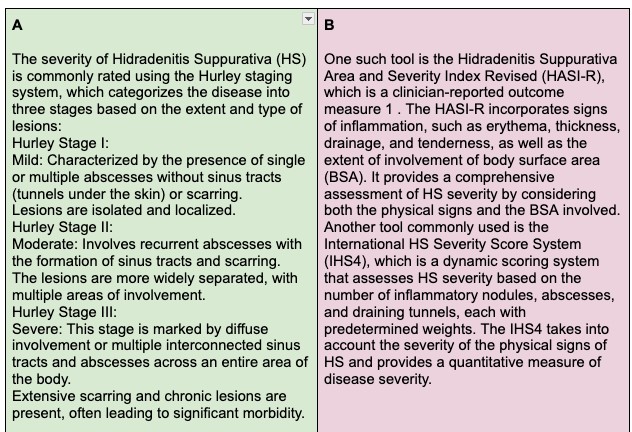


*Check all that apply.*

A is a better response

B is a better response

A and B are about equal in quality of response

I think both answers are inadequate in answering the posed question


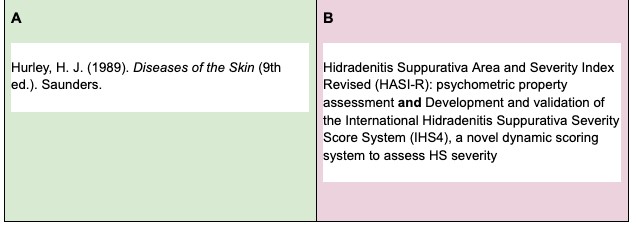


*Check all that apply.*

A has a better reference

B has a better reference

References in A and B are about equal in quality of response

I think both answers are inadequate in answering the posed question

# 24. What margin should be used for lentigo maligna (or severely dysplastic nevus)? *


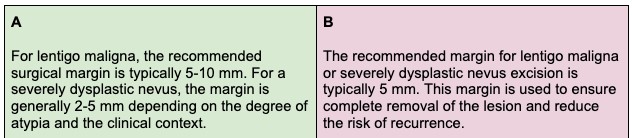


*Check all that apply.*

A is a better response

B is a better response

A and B are about equal in quality of response

I think both answers are inadequate in answering the posed question


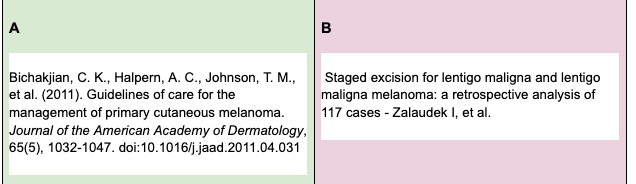


*Check all that apply.*

A has a better reference

B has a better reference

References in A and B are about equal in quality of response

I think both answers are inadequate in answering the posed question

# Which is the best biologic for psoriasis? *


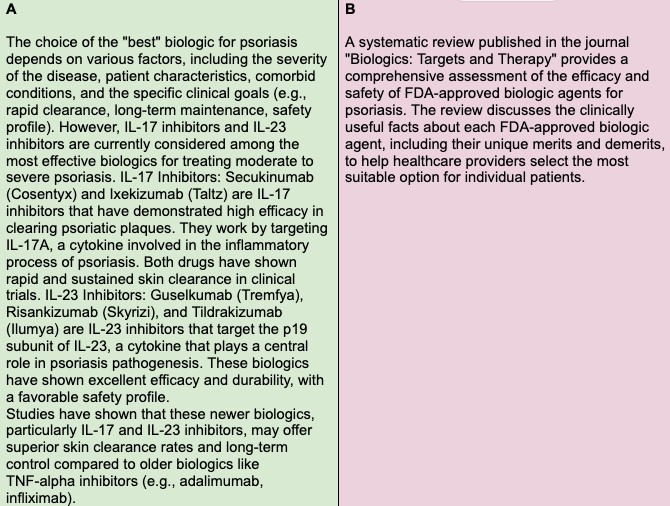


*Check all that apply.*

A is a better response

B is a better response

A and B are about equal in quality of response

I think both answers are inadequate in answering the posed question


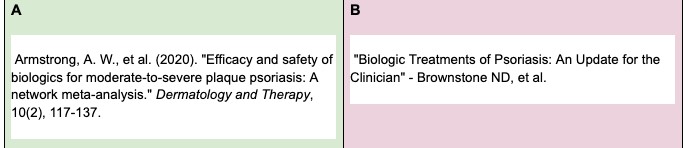


*Check all that apply.*

A has a better reference

B has a better reference

References in A and B are about equal in quality of response

I think both answers are inadequate in answering the posed question

# 28. What is the skin cancer screening recommendation for the general population? *


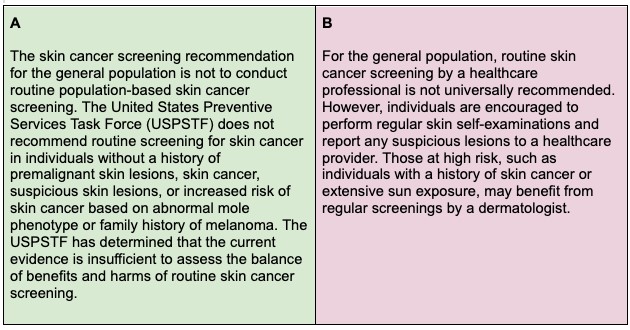


*Check all that apply.*

A is a better response

B is a better response

A and B are about equal in quality of response

I think both answers are inadequate in answering the posed question

29.


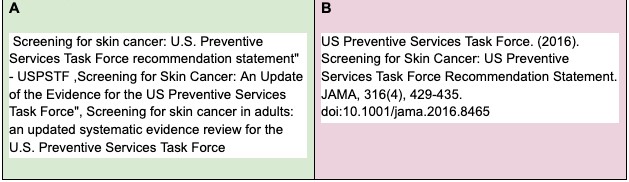


*Check all that apply.*

A has a better reference

B has a better reference

References in A and B are about equal in quality of response

I think both answers are inadequate in answering the posed question

30. **What dermatoses should be included in the differential diagnosis of an eczematous** * **reaction pattern?**


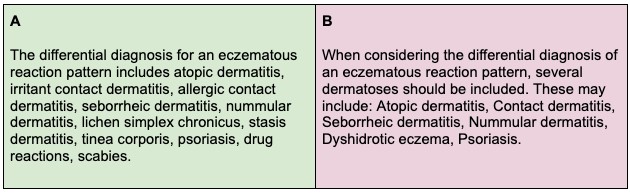


*Check all that apply.*

A is a better response

B is a better response

A and B are about equal in quality of response

I think both answers are inadequate in answering the posed question


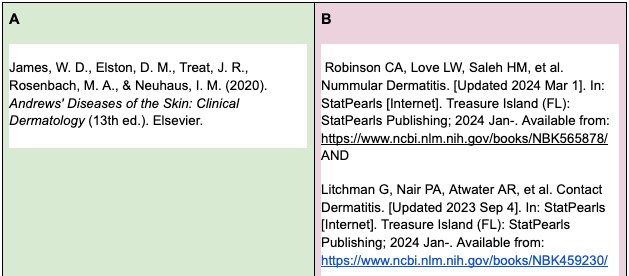


*Check all that apply.*

A has a better reference

B has a better reference

References in A and B are about equal in quality of response

I think both answers are inadequate in answering the posed question

# What medications are used to treat hormonally exacerbated acne? *


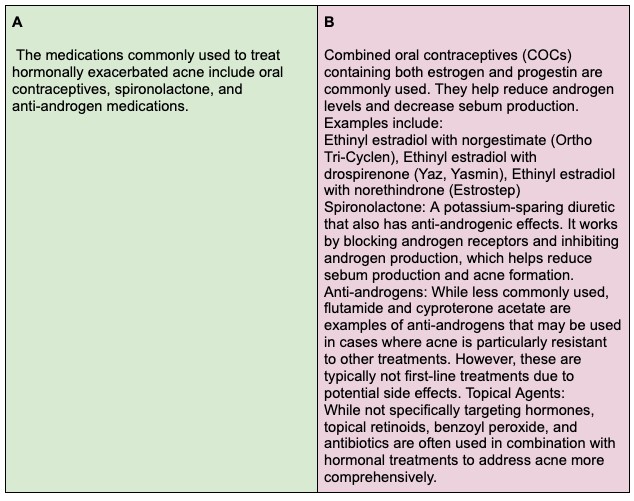


*Check all that apply.*

A is a better response

B is a better response

A and B are about equal in quality of response

I think both answers are inadequate in answering the posed question


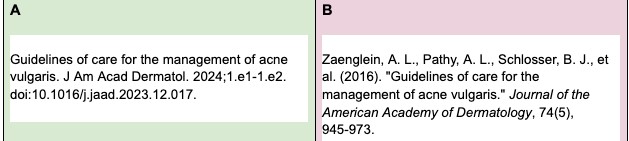


*Check all that apply.*

A has a better reference

B has a better reference

References in A and B are about equal in quality of response

I think both answers are inadequate in answering the posed question

34. Any comments/questions?

This content is neither created nor endorsed by Google.

[Forms](https://www.google.com/forms/about/?utm_source=product&utm_medium=forms_logo&utm_campaign=forms)
