# Supplementary material for: Evaluating Artificial Intelligence Models in Dermatology: Comparative Analysis
Source: JMIR Dermatol. 2025 Dec 4;8:e74040. doi: 10.2196/74040 (PMC12677980; doi:10.2196/74040)
Supplement: Multimedia Appendix 3 [file derma-v8-e74040-s003.docx]

The FREQ Procedure

|  | Q1 |
| --- | --- |
| Q1 | Frequency |
| ChatGPT | 11 |
| DermGPT | 7 |
| Other | 1 |
|  | Q2 |
| Q2 | Frequency |
| ChatGPT | 7 |
| DermGPT | 4 |
| Other | 8 |
|  | Q3 |
| Q3 | Frequency |
| ChatGPT | 12 |
| DermGPT | 5 |
| Other | 2 |
|  | Q4 |
| Q4 | Frequency |
| ChatGPT | 5 |
| DermGPT | 9 |
| Other | 5 |
|  | Q5 |
| Q5 | Frequency |
| ChatGPT | 1 |
| DermGPT | 15 |
| Other | 3 |
|  | Q6 |
| Q6 | Frequency |
| ChatGPT | 3 |
| DermGPT | 4 |
| Other | 12 |
|  | Q7 |
| Q7 | Frequency |
| ChatGPT | 2 |
| DermGPT | 3 |
| Other | 14 |
|  | Q8 |
| Q8 | Frequency |
| ChatGPT | 11 |
|  | Q8 |
| Q8 | Frequency |
| DermGPT | 5 |
| Other | 3 |
|  | Q9 |
| Q9 | Frequency |
| ChatGPT | 6 |
| DermGPT | 9 |
| Other | 4 |
| Q10 | |
| Q10 | Frequency |
| ChatGPT | 14 |
| DermGPT | 1 |
| Other | 4 |
| Q11 | |
| Q11 | Frequency |
| ChatGPT | 3 |
| DermGPT | 16 |
| Q12 | |
| Q12 | Frequency |
| DermGPT | 18 |
| Other | 1 |
| Q13 | |
| Q13 | Frequency |
| ChatGPT | 4 |
| DermGPT | 7 |
| Other | 8 |
| Q14 | |
| Q14 | Frequency |
| DermGPT | 17 |
| Other | 2 |
| Q15 | |
| Q15 | Frequency |
| ChatGPT | 2 |
| DermGPT | 17 |

The FREQ Procedure

Frequency

| Table of Group by Q1 | | | |  |
| --- | --- | --- | --- | --- |
| Group(Group) | Q1(Q1) | | |  |
|  | ChatGPT | DermGPT | Other | Total |
| Attending | 9  47.37 69.23  81.82 | 4  21.05 30.77  57.14 | 0  0.00 0.00  0.00 | 13  68.42 |
| Resident | 2  10.53 33.33  18.18 | 3  15.79 50.00  42.86 | 1  5.26 16.67 100.00 | 6  31.58 |
| Total | 11  57.89 | 7  36.84 | 1  5.26 | 19  100.00 |

Statistics for Table of Group by Q1

| Statistic | DF | Value | Prob |
| --- | --- | --- | --- |
| Chi-Square | 2 | 3.4925 | 0.1744 |
| Likelihood Ratio Chi-Square | 2 | 3.7071 | 0.1567 |
| Mantel-Haenszel Chi-Square | 1 | 3.0306 | 0.0817 |
| Phi Coefficient |  | 0.4287 |  |
| Contingency Coefficient |  | 0.3940 |  |
| Cramer's V |  | 0.4287 |  |
| WARNING: 83% of the cells have expected counts less than 5. Chi-Square may not be a valid test. | | | |

Sample Size = 19

| \| Frequency  Percent Row Pct  Col Pct \| \| --- \| | \| Table of Group by Q2 \| \| \| \|  \| \| --- \| --- \| --- \| --- \| --- \| \| Group(Group) \| Q2(Q2) \| \| \|  \| \| ChatGPT \| DermGPT \| Other \| Total \| \| Attending \| 5  26.32 38.46  71.43 \| 3  15.79 23.08  75.00 \| 5  26.32 38.46  62.50 \| 13  68.42 \| \| Resident \| 2 10.53 33.33  28.57 \| 1 5.26  16.67  25.00 \| 3 15.79 50.00  37.50 \| 6 31.58 \| \| Total \| 7  36.84 \| 4  21.05 \| 8  42.11 \| 19  100.00 \| |
| --- | --- | --- | --- | --- | --- | --- | --- | --- | --- | --- | --- | --- | --- | --- | --- | --- | --- | --- | --- | --- | --- | --- | --- | --- | --- | --- | --- | --- | --- | --- | --- |

Statistics for Table of Group by Q2

| Statistic | DF | Value | Prob |
| --- | --- | --- | --- |
| Chi-Square | 2 | 0.2392 | 0.8873 |
| Likelihood Ratio Chi-Square | 2 | 0.2394 | 0.8872 |
| Mantel-Haenszel Chi-Square | 1 | 0.1373 | 0.7110 |
| Phi Coefficient |  | 0.1122 |  |
| Contingency Coefficient |  | 0.1115 |  |
| Cramer's V |  | 0.1122 |  |
| WARNING: 83% of the cells have expected counts less than 5. Chi-Square may not be a valid test. | | | |

| Table of Group by Q3 | | | |  |
| --- | --- | --- | --- | --- |
| Group(Group) | Q3(Q3) | | |  |
|  | ChatGPT | DermGPT | Other | Total |
| Attending | 7  36.84 53.85  58.33 | 5  26.32  38.46 100.00 | 1  5.26  7.69  50.00 | 13  68.42 |
| Resident | 5  26.32 83.33  41.67 | 0  0.00 0.00  0.00 | 1  5.26 16.67  50.00 | 6  31.58 |
| Total | 12  63.16 | 5  26.32 | 2  10.53 | 19  100.00 |

Statistics for Table of Group by Q3

| Statistic | DF | Value | Prob |
| --- | --- | --- | --- |
| Chi-Square | 2 | 3.1870 | 0.2032 |
| Likelihood Ratio Chi-Square | 2 | 4.6257 | 0.0990 |
| Mantel-Haenszel Chi-Square | 1 | 0.3559 | 0.5508 |
| Phi Coefficient |  | 0.4096 |  |
| Contingency Coefficient |  | 0.3790 |  |
| Cramer's V |  | 0.4096 |  |
| WARNING: 83% of the cells have expected counts less than 5. Chi-Square may not be a valid test. | | | |

Sample Size = 19

| \| Frequency  Percent Row Pct  Col Pct \| \| --- \| | \| Table of Group by Q4 \| \| \| \|  \| \| --- \| --- \| --- \| --- \| --- \| \| Group(Group) \| Q4(Q4) \| \| \|  \| \| ChatGPT \| DermGPT \| Other \| Total \| \| Attending \| 5  26.32 38.46 100.00 \| 4  21.05 30.77  44.44 \| 4  21.05 30.77  80.00 \| 13  68.42 \| \| Resident \| 0 0.00 0.00  0.00 \| 5 26.32 83.33  55.56 \| 1 5.26  16.67  20.00 \| 6 31.58 \| \| Total \| 5  26.32 \| 9  47.37 \| 5  26.32 \| 19  100.00 \| |
| --- | --- | --- | --- | --- | --- | --- | --- | --- | --- | --- | --- | --- | --- | --- | --- | --- | --- | --- | --- | --- | --- | --- | --- | --- | --- | --- | --- | --- | --- | --- | --- |

Statistics for Table of Group by Q4

| Statistic | | DF | Value | Prob |  |
| --- | --- | --- | --- | --- | --- |
| Chi-Square | | 2 | 5.0125 | 0.0816 |  |
| Likelihood Ratio Chi-Square | | 2 | 6.3296 | 0.0422 |  |
| Mantel-Haenszel Chi-Square | | 1 | 0.4385 | 0.5079 |  |
| Phi Coefficient | |  | 0.5136 |  |  |
| Contingency Coefficient | |  | 0.4569 |  |  |
| Cramer's V | |  | 0.5136 |  |  |
| WARNING: 83% of the cells have expected counts less than 5. Chi-Square may not be a valid test. | | | | |  |
| Table of Group by Q5 | | | | |  |
| Group(Group) | Q5(Q5) | | | |  |
|  | ChatGPT | DermGPT | | Other | Total |
| Attending | 1  5.26  7.69 100.00 | 9  47.37 69.23  60.00 | | 3  15.79  23.08 100.00 | 13  68.42 |
| Resident | 0  0.00 0.00  0.00 | 6  31.58 100.00  40.00 | | 0  0.00 0.00  0.00 | 6  31.58 |
| Total | 1  5.26 | 15  78.95 | | 3  15.79 | 19  100.00 |

Statistics for Table of Group by Q5

| Statistic | DF | Value | Prob |
| --- | --- | --- | --- |
| Chi-Square | 2 | 2.3385 | 0.3106 |
| Likelihood Ratio Chi-Square | 2 | 3.5085 | 0.1730 |
| Mantel-Haenszel Chi-Square | 1 | 0.4615 | 0.4969 |
| Phi Coefficient |  | 0.3508 |  |
| Contingency Coefficient |  | 0.3310 |  |
| Cramer's V |  | 0.3508 |  |
| WARNING: 83% of the cells have expected counts less than 5. Chi-Square may not be a valid test. | | | |

Sample Size = 19

| \| Frequency  Percent Row Pct  Col Pct \| \| --- \| | \| Table of Group by Q6 \| \| \| \|  \| \| --- \| --- \| --- \| --- \| --- \| \| Group(Group) \| Q6(Q6) \| \| \|  \| \| ChatGPT \| DermGPT \| Other \| Total \| \| Attending \| 1  5.26 7.69  33.33 \| 4  21.05 30.77 100.00 \| 8 42.11 61.54  66.67 \| 13  68.42 \| \| Resident \| 2 10.53 33.33  66.67 \| 0 0.00 0.00  0.00 \| 4 21.05 66.67  33.33 \| 6 31.58 \| \| Total \| 3  15.79 \| 4  21.05 \| 12  63.16 \| 19  100.00 \| |
| --- | --- | --- | --- | --- | --- | --- | --- | --- | --- | --- | --- | --- | --- | --- | --- | --- | --- | --- | --- | --- | --- | --- | --- | --- | --- | --- | --- | --- | --- | --- | --- |

Statistics for Table of Group by Q6

| Statistic | | DF | Value | Prob |  |
| --- | --- | --- | --- | --- | --- |
| Chi-Square | | 2 | 3.5726 | 0.1676 |  |
| Likelihood Ratio Chi-Square | | 2 | 4.6035 | 0.1001 |  |
| Mantel-Haenszel Chi-Square | | 1 | 0.2896 | 0.5905 |  |
| Phi Coefficient | |  | 0.4336 |  |  |
| Contingency Coefficient | |  | 0.3978 |  |  |
| Cramer's V | |  | 0.4336 |  |  |
| WARNING: 83% of the cells have expected counts less than 5. Chi-Square may not be a valid test. | | | | |  |
| Table of Group by Q7 | | | | |  |
| Group(Group) | Q7(Q7) | | | |  |
|  | ChatGPT | DermGPT | | Other | Total |
| Attending | 2  10.53  15.38 100.00 | 3  15.79  23.08 100.00 | | 8 42.11  61.54  57.14 | 13  68.42 |
| Resident | 0  0.00 0.00  0.00 | 0  0.00 0.00  0.00 | | 6  31.58 100.00  42.86 | 6  31.58 |
| Total | 2  10.53 | 3  15.79 | | 14  73.68 | 19  100.00 |

Statistics for Table of Group by Q7

| Statistic | DF | Value | Prob |
| --- | --- | --- | --- |
| Chi-Square | 2 | 3.1319 | 0.2089 |
| Likelihood Ratio Chi-Square | 2 | 4.5775 | 0.1014 |
| Mantel-Haenszel Chi-Square | 1 | 2.5442 | 0.1107 |
| Phi Coefficient |  | 0.4060 |  |
| Contingency Coefficient |  | 0.3762 |  |
| Cramer's V |  | 0.4060 |  |
| WARNING: 83% of the cells have expected counts less than 5. Chi-Square may not be a valid test. | | | |

Sample Size = 19

| \| Frequency  Percent Row Pct  Col Pct \| \| --- \| | \| Table of Group by Q8 \| \| \| \|  \| \| --- \| --- \| --- \| --- \| --- \| \| Group(Group) \| Q8(Q8) \| \| \|  \| \| ChatGPT \| DermGPT \| Other \| Total \| \| Attending \| 5  26.32 38.46  45.45 \| 5  26.32 38.46 100.00 \| 3  15.79 23.08 100.00 \| 13  68.42 \| \| Resident \| 6 31.58  100.00  54.55 \| 0 0.00 0.00  0.00 \| 0 0.00 0.00  0.00 \| 6 31.58 \| \| Total \| 11  57.89 \| 5  26.32 \| 3  15.79 \| 19  100.00 \| |
| --- | --- | --- | --- | --- | --- | --- | --- | --- | --- | --- | --- | --- | --- | --- | --- | --- | --- | --- | --- | --- | --- | --- | --- | --- | --- | --- | --- | --- | --- | --- | --- |

Statistics for Table of Group by Q8

| Statistic | DF | Value | Prob |
| --- | --- | --- | --- |
| Chi-Square | 2 | 6.3776 | 0.0412 |
| Likelihood Ratio Chi-Square | 2 | 8.5407 | 0.0140 |
| Mantel-Haenszel Chi-Square | 1 | 4.9764 | 0.0257 |
| Phi Coefficient |  | 0.5794 |  |
| Contingency Coefficient |  | 0.5013 |  |
| Cramer's V |  | 0.5794 |  |
| WARNING: 83% of the cells have expected counts less than 5. Chi-Square may not be a valid test. | | | |

| Table of Group by Q9 | | | |  |
| --- | --- | --- | --- | --- |
| Group(Group) | Q9(Q9) | | |  |
|  | ChatGPT | DermGPT | Other | Total |
| Attending | 6  31.58  46.15 100.00 | 5  26.32 38.46  55.56 | 2  10.53 15.38  50.00 | 13  68.42 |
| Resident | 0  0.00 0.00  0.00 | 4  21.05 66.67  44.44 | 2  10.53 33.33  50.00 | 6  31.58 |
| Total | 6  31.58 | 9  47.37 | 4  21.05 | 19  100.00 |

Statistics for Table of Group by Q9

| Statistic | DF | Value | Prob |
| --- | --- | --- | --- |
| Chi-Square | 2 | 4.0869 | 0.1296 |
| Likelihood Ratio Chi-Square | 2 | 5.7884 | 0.0553 |
| Mantel-Haenszel Chi-Square | 1 | 3.1017 | 0.0782 |
| Phi Coefficient |  | 0.4638 |  |
| Contingency Coefficient |  | 0.4207 |  |
| Cramer's V |  | 0.4638 |  |
| WARNING: 83% of the cells have expected counts less than 5. Chi-Square may not be a valid test. | | | |

Sample Size = 19

| \| Frequency  Percent Row Pct  Col Pct \| \| --- \| | \| Table of Group by Q10 \| \| \| \|  \| \| --- \| --- \| --- \| --- \| --- \| \| Group(Group) \| Q10(Q10) \| \| \|  \| \| ChatGPT \| DermGPT \| Other \| Total \| \| Attending \| 8 42.11 61.54  57.14 \| 1  5.26 7.69 100.00 \| 4  21.05 30.77 100.00 \| 13  68.42 \| \| Resident \| 6 31.58  100.00  42.86 \| 0 0.00 0.00  0.00 \| 0 0.00 0.00  0.00 \| 6 31.58 \| \| Total \| 14  73.68 \| 1  5.26 \| 4  21.05 \| 19  100.00 \| |
| --- | --- | --- | --- | --- | --- | --- | --- | --- | --- | --- | --- | --- | --- | --- | --- | --- | --- | --- | --- | --- | --- | --- | --- | --- | --- | --- | --- | --- | --- | --- | --- |

Statistics for Table of Group by Q10

| Statistic | DF | Value | Prob |
| --- | --- | --- | --- |
| Chi-Square | 2 | 3.1319 | 0.2089 |
| Likelihood Ratio Chi-Square | 2 | 4.5775 | 0.1014 |
| Mantel-Haenszel Chi-Square | 1 | 2.7807 | 0.0954 |
| Phi Coefficient |  | 0.4060 |  |
| Contingency Coefficient |  | 0.3762 |  |
| Cramer's V |  | 0.4060 |  |
| WARNING: 83% of the cells have expected counts less than 5. Chi-Square may not be a valid test. | | | |

Frequency

| Table of Group by Q11 | | |  |
| --- | --- | --- | --- |
| Group(Group) | Q11(Q11) | |  |
|  | ChatGPT | DermGPT | Total |
| Attending | 2  10.53 15.38  66.67 | 11  57.89 84.62  68.75 | 13  68.42 |
| Resident | 1  5.26 16.67  33.33 | 5  26.32 83.33  31.25 | 6  31.58 |
| Total | 3  15.79 | 16  84.21 | 19  100.00 |

| Percent  Row Pct  Col Pct |
| --- |

Statistics for Table of Group by Q11

| Statistic | | DF | | Value | | Prob |
| --- | --- | --- | --- | --- | --- | --- |
| Chi-Square | | 1 | | 0.0051 | | 0.9432 |
| Likelihood Ratio Chi-Square | | 1 | | 0.0050 | | 0.9434 |
| Continuity Adj. Chi-Square | | 1 | | 0.0000 | | 1.0000 |
| Mantel-Haenszel Chi-Square | | 1 | | 0.0048 | | 0.9447 |
| Phi Coefficient | |  | | -0.0163 | |  |
| Contingency Coefficient | |  | | 0.0163 | |  |
| Cramer's V | |  | | -0.0163 | |  |
| WARNING: 50% of the cells have expected counts less than 5. Chi-Square may not be a valid test. | | | | | | |
| Fisher's Exact Test | | | |  |  |  |
| Cell (1,1) Frequency (F) | | 2 | |  |  |  |
| Left-sided Pr <= F | | 0.7049 | |  |  |  |
| Right-sided Pr >= F | | 0.7781 | |  |  |  |
|  | |  | |  |  |  |
| Table Probability (P) | | 0.4830 | |  |  |  |
| Two-sided Pr <= P | | 1.0000 | |  |  |  |

Sample Size = 19

| \| Frequency  Percent  Row Pct  Col Pct \| \| --- \| | \| Table of Group by Q12 \| \| \|  \| \| --- \| --- \| --- \| --- \| \| Group(Group) \| Q12(Q12) \| \|  \| \| DermGPT \| Other \| Total \| \| Attending \| 12 63.16 92.31  66.67 \| 1 5.26  7.69 100.00 \| 13 68.42 \| \| Resident \| 6  31.58  100.00  33.33 \| 0  0.00 0.00  0.00 \| 6  31.58 \| \| Total \| 18  94.74 \| 1  5.26 \| 19  100.00 \| |
| --- | --- | --- | --- | --- | --- | --- | --- | --- | --- | --- | --- | --- | --- | --- | --- | --- | --- | --- | --- | --- | --- | --- | --- | --- | --- |

Statistics for Table of Group by Q12

| Statistic | | DF | | Value | | Prob |
| --- | --- | --- | --- | --- | --- | --- |
| Chi-Square | | 1 | | 0.4872 | | 0.4852 |
| WARNING: 50% of the cells have expected counts less than 5. Chi-Square may not be a valid test. | | | | | | |
| Fisher's Exact Test | | | |  |  |  |
| Cell (1,1) Frequency (F) | | 12 | |  |  |  |
| Left-sided Pr <= F | | 0.6842 | |  |  |  |
| Right-sided Pr >= F | | 1.0000 | |  |  |  |
|  | |  | |  |  |  |
| Table Probability (P) | | 0.6842 | |  |  |  |
| Two-sided Pr <= P | | 1.0000 | |  |  |  |

| Statistic | DF | Value | Prob |
| --- | --- | --- | --- |
| Likelihood Ratio Chi-Square | 1 | 0.7844 | 0.3758 |
| Continuity Adj. Chi-Square | 1 | 0.0000 | 1.0000 |
| Mantel-Haenszel Chi-Square | 1 | 0.4615 | 0.4969 |
| Phi Coefficient |  | -0.1601 |  |
| Contingency Coefficient |  | 0.1581 |  |
| Cramer's V |  | -0.1601 |  |
| WARNING: 50% of the cells have expected counts less than 5. Chi-Square may not be a valid test. | | | |

Sample Size = 19

| \| Frequency  Percent Row Pct  Col Pct \| \| --- \| | \| Table of Group by Q13 \| \| \| \|  \| \| --- \| --- \| --- \| --- \| --- \| \| Group(Group) \| Q13(Q13) \| \| \|  \| \| ChatGPT \| DermGPT \| Other \| Total \| \| Attending \| 3  15.79 23.08  75.00 \| 5  26.32 38.46  71.43 \| 5  26.32 38.46  62.50 \| 13  68.42 \| \| Resident \| 1  5.26 16.67  25.00 \| 2  10.53 33.33  28.57 \| 3  15.79 50.00  37.50 \| 6  31.58 \| \| Total \| 4  21.05 \| 7  36.84 \| 8  42.11 \| 19  100.00 \| |
| --- | --- | --- | --- | --- | --- | --- | --- | --- | --- | --- | --- | --- | --- | --- | --- | --- | --- | --- | --- | --- | --- | --- | --- | --- | --- | --- | --- | --- | --- | --- | --- |

Statistics for Table of Group by Q13

| Statistic | DF | Value | Prob |
| --- | --- | --- | --- |
| Chi-Square | 2 | 0.2392 | 0.8873 |
| Likelihood Ratio Chi-Square | 2 | 0.2394 | 0.8872 |
| Mantel-Haenszel Chi-Square | 1 | 0.2134 | 0.6442 |
| Phi Coefficient |  | 0.1122 |  |
| Contingency Coefficient |  | 0.1115 |  |
| Cramer's V |  | 0.1122 |  |
| WARNING: 83% of the cells have expected counts less than 5. Chi-Square may not be a valid test. | | | |

Sample Size = 19

| \| Frequency  Percent Row Pct  Col Pct \| \| --- \| | \| Table of Group by Q14 \| \| \|  \| \| --- \| --- \| --- \| --- \| \| Group(Group) \| Q14(Q14) \| \|  \| \| DermGPT \| Other \| Total \| \| Attending \| 11  57.89 84.62  64.71 \| 2  10.53 15.38 100.00 \| 13  68.42 \| |
| --- | --- | --- | --- | --- | --- | --- | --- | --- | --- | --- | --- | --- | --- | --- | --- | --- | --- |

| Table of Group by Q14 | | |  |
| --- | --- | --- | --- |
| Group(Group) | Q14(Q14) | |  |
|  | DermGPT | Other | Total |
| Resident | 6  31.58  100.00  35.29 | 0  0.00 0.00  0.00 | 6  31.58 |
| Total | 17  89.47 | 2  10.53 | 19  100.00 |

Statistics for Table of Group by Q14

| Statistic | | DF | | Value | | Prob |
| --- | --- | --- | --- | --- | --- | --- |
| Chi-Square | | 1 | | 1.0317 | | 0.3098 |
| Likelihood Ratio Chi-Square | | 1 | | 1.6244 | | 0.2025 |
| Continuity Adj. Chi-Square | | 1 | | 0.0448 | | 0.8324 |
| Mantel-Haenszel Chi-Square | | 1 | | 0.9774 | | 0.3228 |
| Phi Coefficient | |  | | -0.2330 | |  |
| Contingency Coefficient | |  | | 0.2269 | |  |
| Cramer's V | |  | | -0.2330 | |  |
| WARNING: 50% of the cells have expected counts less than 5. Chi-Square may not be a valid test. | | | | | | |
| Fisher's Exact Test | | | |  |  |  |
| Cell (1,1) Frequency (F) | | 11 | |  |  |  |
| Left-sided Pr <= F | | 0.4561 | |  |  |  |
| Right-sided Pr >= F | | 1.0000 | |  |  |  |
|  | |  | |  |  |  |
| Table Probability (P) | | 0.4561 | |  |  |  |
| Two-sided Pr <= P | | 1.0000 | |  |  |  |

Sample Size = 19

| \| Frequency  Percent  Row Pct  Col Pct \| \| --- \| | \| Table of Group by Q15 \| \| \|  \| \| --- \| --- \| --- \| --- \| \| Group(Group) \| Q15(Q15) \| \|  \| \| ChatGPT \| DermGPT \| Total \| \| Attending \| 2 10.53  15.38 100.00 \| 11 57.89 84.62  64.71 \| 13 68.42 \| \| Resident \| 0 0.00 0.00  0.00 \| 6 31.58  100.00  35.29 \| 6 31.58 \| \| Total \| 2  10.53 \| 17  89.47 \| 19  100.00 \| |
| --- | --- | --- | --- | --- | --- | --- | --- | --- | --- | --- | --- | --- | --- | --- | --- | --- | --- | --- | --- | --- | --- | --- | --- | --- | --- |

Statistics for Table of Group by Q15

| Statistic | | DF | | Value | | Prob |
| --- | --- | --- | --- | --- | --- | --- |
| Chi-Square | | 1 | | 1.0317 | | 0.3098 |
| Likelihood Ratio Chi-Square | | 1 | | 1.6244 | | 0.2025 |
| Continuity Adj. Chi-Square | | 1 | | 0.0448 | | 0.8324 |
| WARNING: 50% of the cells have expected counts less than 5. Chi-Square may not be a valid test. | | | | | | |
| Fisher's Exact Test | | | |  |  |  |
| Cell (1,1) Frequency (F) | | 2 | |  |  |  |
| Left-sided Pr <= F | | 1.0000 | |  |  |  |
| Right-sided Pr >= F | | 0.4561 | |  |  |  |
|  | |  | |  |  |  |
| Table Probability (P) | | 0.4561 | |  |  |  |
| Two-sided Pr <= P | | 1.0000 | |  |  |  |

| Statistic | DF | Value | Prob |
| --- | --- | --- | --- |
| Mantel-Haenszel Chi-Square | 1 | 0.9774 | 0.3228 |
| Phi Coefficient |  | 0.2330 |  |
| Contingency Coefficient |  | 0.2269 |  |
| Cramer's V |  | 0.2330 |  |
| WARNING: 50% of the cells have expected counts less than 5. Chi-Square may not be a valid test. | | | |

Sample Size = 19
