# Supplementary material for: Evaluating Artificial Intelligence Models in Dermatology: Comparative Analysis
Source: JMIR Dermatol. 2025 Dec 4;8:e74040. doi: 10.2196/74040 (PMC12677980; doi:10.2196/74040)
Supplement: Multimedia Appendix 4 [file derma-v8-e74040-s004.docx]

12/29/24, 9:26 PM Results: sourcedata.sas

**The FREQ Procedure**

| \| **Frequency**  **Percent**  **Row Pct**  **Col Pct** \| \| --- \| | \| **Table of Group by Response** \| \| \| \|  \| \| --- \| --- \| --- \| --- \| --- \| \| **Group(Group)** \| **Response(Response)** \| \| \|  \| \| **ChatGPT** \| **DermGPT** \| **Other** \| **Total** \| \| **Attending** \| 94  32.98 48.21  71.76 \| 45  15.79 23.08  67.16 \| 56  19.65 28.72  64.37 \| 195  68.42 \| \| **Resident** \| 37 12.98 41.11  28.24 \| 22 7.72  24.44  32.84 \| 31 10.88 34.44  35.63 \| 90 31.58 \| \| **Total** \| 131  45.96 \| 67  23.51 \| 87  30.53 \| 285  100.00 \| |
| --- | --- | --- | --- | --- | --- | --- | --- | --- | --- | --- | --- | --- | --- | --- | --- | --- | --- | --- | --- | --- | --- | --- | --- | --- | --- | --- | --- | --- | --- | --- | --- |

**Statistics for Table of Group by Response**

| **Statistic** | **DF** | **Value** | **Prob** |
| --- | --- | --- | --- |
| **Chi-Square** | 2 | 1.3847 | 0.5004 |
| **Likelihood Ratio Chi-Square** | 2 | 1.3842 | 0.5005 |
| **Mantel-Haenszel Chi-Square** | 1 | 1.3610 | 0.2434 |
| **Phi Coefficient** |  | 0.0697 |  |
| **Contingency Coefficient** |  | 0.0695 |  |
| **Cramer's V** |  | 0.0697 |  |

**Sample Size = 285**

https://odamid-usw2-2.oda.sas.com/SASStudio/sasexec/submissions/c368daaf-04e8-4531-a1ea-180fdaa46f49/results 1/1
