# Supplementary material for: Evaluating Artificial Intelligence Models in Dermatology: Comparative Analysis
Source: JMIR Dermatol. 2025 Dec 4;8:e74040. doi: 10.2196/74040 (PMC12677980; doi:10.2196/74040)
Supplement: Multimedia Appendix 5 [file derma-v8-e74040-s005.docx]

12/29/24, 6:02 PM Results: longdataset.sas

**The FREQ Procedure**

| \| **Frequency**  **Percent**  **Row Pct**  **Col Pct** \| \| --- \| | \| **Table of Group by Response** \| \| \| \|  \| \| --- \| --- \| --- \| --- \| --- \| \| **Group(Group)** \| **Response(Response)** \| \| \|  \| \| **ChatGPT** \| **DermGPT** \| **Other** \| **Total** \| \| **Attending** \| 56  19.65 28.72  69.14 \| 93  32.63 47.69  67.88 \| 46  16.14 23.59  68.66 \| 195  68.42 \| \| **Resident** \| 25 8.77  27.78  30.86 \| 44 15.44 48.89  32.12 \| 21 7.37  23.33  31.34 \| 90 31.58 \| \| **Total** \| 81  28.42 \| 137  48.07 \| 67  23.51 \| 285  100.00 \| |
| --- | --- | --- | --- | --- | --- | --- | --- | --- | --- | --- | --- | --- | --- | --- | --- | --- | --- | --- | --- | --- | --- | --- | --- | --- | --- | --- | --- | --- | --- | --- | --- |

**Statistics for Table of Group by Response**

| **Statistic** | **DF** | **Value** | **Prob** |
| --- | --- | --- | --- |
| **Chi-Square** | 2 | 0.0392 | 0.9806 |
| **Likelihood Ratio Chi-Square** | 2 | 0.0392 | 0.9806 |
| **Mantel-Haenszel Chi-Square** | 1 | 0.0056 | 0.9406 |
| **Phi Coefficient** |  | 0.0117 |  |
| **Contingency Coefficient** |  | 0.0117 |  |
| **Cramer's V** |  | 0.0117 |  |

**Sample Size = 285**

https://odamid-usw2-2.oda.sas.com/SASStudio/sasexec/submissions/b994fd42-664f-49a6-9225-64261c541a22/results 1/1
