# Supplementary material for: Evaluating Artificial Intelligence Models in Dermatology: Comparative Analysis
Source: JMIR Dermatol. 2025 Dec 4;8:e74040. doi: 10.2196/74040 (PMC12677980; doi:10.2196/74040)
Supplement: Multimedia Appendix 6 [file derma-v8-e74040-s006.docx]

*Prompt: Please answer this question about dermatology and provide a source from a scientific journal or another authoritative source.*

*First answer in all text: ChatGPT followed by DermGPT for answers below, randomized using the list*

*Model:* ***Chat GPT-4o; new account; questions September 3 10:00AM***

*Answer boxes using randomizer tool (https://www.random.org/lists/):*

1. *R chatgpt (B)*
2. *R dermgpt (B)*
3. *L dermgpt*
4. *L chatgpt*
5. *R chatgpt*
6. *L chatgpt*
7. *R dermgpt*
8. *L dermgpt*
9. *R chatgpt*
10. *L dermgpt*
11. *L chatgpt*
12. *R dermgpt*
13. *R chatgpt*
14. *L chatgpt*
15. *L dermgpt.*

**1. What is the best treatment for mild psoriasis?**

**Answer (ChatGPT)**: Common Treatments for Mild Psoriasis:

1. **Topical Corticosteroids**: These are the most commonly used treatments for mild psoriasis. They help reduce inflammation and slow down the rapid turnover of skin cells. Low to medium potency corticosteroids are usually recommended for most cases of mild psoriasis.
2. **Vitamin D Analogues (e.g., Calcipotriene)**: These compounds help to slow the growth of skin cells. They are often used in combination with corticosteroids to enhance effectiveness.
3. **Topical Retinoids (e.g., Tazarotene)**: Retinoids help normalize skin cell production and are effective in treating plaque psoriasis. However, they can cause irritation, so they are often used in combination with corticosteroids.
4. **Moisturizers and Emollients**: Regular use of moisturizers can help reduce dryness, scaling, and itching, which are common symptoms of psoriasis.

**Source**: **Journal of the American Academy of Dermatology (JAAD)**: Provides comprehensive guidelines on the management of psoriasis, including the use of topical therapies as a first-line treatment for mild cases ***AND*** **Dermatology: Therapy and Practice (Springer)**: Discusses various treatment options for psoriasis, highlighting the efficacy of topical agents in managing mild cases.

Answer (DermGPT): The best treatment for mild psoriasis can vary depending on the individual and their specific symptoms. However, topical therapies are often recommended as the first-line treatment for mild psoriasis . These may include corticosteroids, vitamin D analogs, retinoids, or coal tar preparations. Topical corticosteroids, in particular, are commonly used and have been shown to effectively reduce inflammation and relieve symptoms associated with mild psoriasis. It is important to note that treatment decisions should be made in consultation with a dermatologist who can assess the severity of the condition and tailor the treatment plan accordingly.

Source: Armstrong AW, Koning JW, Rowse S, Tan H, Mamolo C, Kaur M. Under-Treatment of Patients with Moderate to Severe Psoriasis in the United States: Analysis of Medication Usage with Health Plan Data. Dermatol Ther (Heidelb). 2017 Mar;7(1):97-109. doi: 10.1007/s13555-016-0153-2. Epub 2016 Nov 30. PMID: 27905011; PMCID: PMC5336424.

| **A**  The best treatment for mild psoriasis can vary depending on the individual and their specific symptoms. However, topical therapies are often recommended as the first-line treatment for mild psoriasis . These may include corticosteroids, vitamin D analogs, retinoids, or coal tar preparations. Topical corticosteroids, in particular, are commonly used and have been shown to effectively reduce inflammation and relieve symptoms associated with mild psoriasis. It is important to note that treatment decisions should be made in consultation with a dermatologist who can assess the severity of the condition and tailor the treatment plan accordingly.  **A**  Armstrong AW, Koning JW, Rowse S, Tan H, Mamolo C, Kaur M. Under-Treatment of Patients with Moderate to Severe Psoriasis in the United States: Analysis of Medication Usage with Health Plan Data. Dermatol Ther (Heidelb). 2017 Mar;7(1):97-109. doi: 10.1007/s13555-016-0153-2. Epub 2016 Nov 30. PMID: 27905011; PMCID: PMC5336424. | **B**  Topical Corticosteroids: These are the most commonly used treatments for mild psoriasis. They help reduce inflammation and slow down the rapid turnover of skin cells. Low to medium potency corticosteroids are usually recommended for most cases of mild psoriasis. Vitamin D Analogues (e.g., Calcipotriene): These compounds help to slow the growth of skin cells. They are often used in combination with corticosteroids to enhance effectiveness. Topical Retinoids (e.g., Tazarotene): Retinoids help normalize skin cell production and are effective in treating plaque psoriasis. However, they can cause irritation, so they are often used in combination with corticosteroids. Moisturizers and Emollients: Regular use of moisturizers can help reduce dryness, scaling, and itching, which are common symptoms of psoriasis.  **B**  Journal of the American Academy of Dermatology (JAAD  Dermatology: Therapy and Practice (Springer) |
| --- | --- |

**2. What is the difference between a papule and a macule?**

**Answer (ChatGPT)**: A **papule** and a **macule** are both types of skin lesions, but they differ in their physical characteristics.

- **Papule**: A papule is a small, raised lesion on the skin, typically less than 1 centimeter in diameter. It is palpable and can be of various shapes such as dome-shaped, flat-topped, or pointed. Papules are caused by a variety of factors, including inflammation, infection, or other skin conditions such as psoriasis, acne, or eczema.
- **Macule**: A macule, on the other hand, is a flat, distinct, discolored area of skin that is not raised or depressed compared to the surrounding skin. It is usually less than 1 centimeter in diameter and can be any color, but common colors include red, brown, or white. Macules are typically associated with conditions like freckles, vitiligo, or drug reactions.

### **Key Differences:**

- **Elevation**: Papules are raised, whereas macules are flat.
- **Palpability**: Papules are palpable, but macules are not.
- **Size**: Both are generally small, usually less than 1 cm, but this is not a defining feature for differentiation.

**Source**: Habif, T. P. (2015). *Clinical Dermatology: A Color Guide to Diagnosis and Therapy* (6th ed.). Elsevier.

Answer (DermGPT): A papule is a small, elevated lesion that measures less than 1 centimeter in diameter. It is typically solid and palpable, meaning it can be felt when touched. Papules can have various colors, such as red, pink, brown, or flesh-colored. They may be caused by inflammation, infection, or the accumulation of cells or substances in the skin .A macule is a flat, discolored lesion that is less than 1 centimeter in diameter. It does not have any palpable elevation or change in texture compared to the surrounding skin. Macules can have different colors, such as red, brown, white, or tan. They are caused by changes in the pigment-producing cells in the skin or by the accumulation of substances in the skin. In summary, the main difference between a papule and a macule is that a papule is a small, elevated lesion, while a macule is a flat, discolored lesion.

Source: : "Clinical and dermoscopic features of cutaneous BAP1-inactivated melanocytic tumors: Results of a multicenter case-control study by the International Dermoscopy Society" by Oriol Yélamos et al. (2019)

1 : "Dome-shaped papules with pink-to-tan structureless areas and peripheral irregular dots/globules or network should raise the clinical suspicion for BIMT" by Oriol Yélamos et al. (2019) "Sun protection" by David Leffell (2015)

| **A**  A papule and a macule are both types of skin lesions, but they differ in their physical characteristics. A papule is a small, raised lesion on the skin, typically less than 1 centimeter in diameter. It is palpable and can be of various shapes such as dome-shaped, flat-topped, or pointed. Papules are caused by a variety of factors, including inflammation, infection, or other skin conditions such as psoriasis, acne, or eczema. Macule: A macule, on the other hand, is a flat, distinct, discolored area of skin that is not raised or depressed compared to the surrounding skin. It is usually less than 1 centimeter in diameter and can be any color, but common colors include red, brown, or white. Macules are typically associated with conditions like freckles, vitiligo, or drug reactions.  **A**  Habif, T. P. (2015). *Clinical Dermatology: A Color Guide to Diagnosis and Therapy* (6th ed.). Elsevier. | **B**  A papule is a small, elevated lesion that measures less than 1 centimeter in diameter. It is typically solid and palpable, meaning it can be felt when touched. Papules can have various colors, such as red, pink, brown, or flesh-colored. They may be caused by inflammation, infection, or the accumulation of cells or substances in the skin. A macule is a flat, discolored lesion that is less than 1 centimeter in diameter. It does not have any palpable elevation or change in texture compared to the surrounding skin. Macules can have different colors, such as red, brown, white, or tan. They are caused by changes in the pigment-producing cells in the skin or by the accumulation of substances in the skin. In summary, the main difference between a papule and a macule is that a papule is a small, elevated lesion, while a macule is a flat, discolored lesion.  **B**  "Clinical and dermoscopic features of cutaneous BAP1-inactivated melanocytic tumors: Results of a multicenter case-control study by the International Dermoscopy Society" by Oriol Yélamos et al. (2019) |
| --- | --- |

**3. What are the most reported side effects of IL-23 inhibitors?**

**Answer (ChatGPT)**: The most commonly reported side effects of IL-23 inhibitors, which are used to treat conditions like psoriasis, include:

- **Upper Respiratory Tract Infections**: These are the most frequently observed side effects, often presenting as mild infections such as the common cold.
- **Headache**: Another common side effect reported by patients using IL-23 inhibitors.
- **Injection Site Reactions**: Patients often experience mild to moderate reactions at the site of the injection, including redness, swelling, or pain.
- **Fatigue**: Some individuals report feeling unusually tired after using these medications.
- **Gastrointestinal Issues**: Nausea, diarrhea, and other gastrointestinal symptoms have been reported, though they tend to be mild.

IL-23 inhibitors are generally well-tolerated, but more serious adverse effects, though rare, can include the risk of serious infections and hypersensitivity reactions. Monitoring is essential, especially during long-term use.

**Source**: Blauvelt, A., Papp, K. A., Griffiths, C. E. M., et al. (2017). Efficacy and safety of guselkumab, an interleukin-23–specific monoclonal antibody, compared with adalimumab for the treatment of moderate to severe psoriasis. *Lancet*, 390(10091), 228-238. doi:10.1016/S0140-6736(17)31216-3

**Answer (DermGPT)**: The most commonly reported side effects of IL-23 inhibitors include headaches, conjunctivitis, keratitis, blepharitis, nasopharyngitis, and injection-site reactions.

Source: Dupilumab as a Therapeutic Approach in Alopecia Universalis. Cutis. 2022 November;110(5):E7-E8 | doi:10.12788/cutis.0659. Michael J. Visconti, DO Adam Richardson, DO Ann A. LaFond, MD

| **A**  The most commonly reported side effects of IL-23 inhibitors include headaches, conjunctivitis, keratitis, blepharitis, nasopharyngitis, and injection-site reactions  **A**  Dupilumab as a Therapeutic Approach in Alopecia Universalis. Cutis. 2022 November;110(5):E7-E8 \| doi:10.12788/cutis.0659. Michael J. Visconti, DO Adam Richardson, DO Ann A. LaFond, MD | **B**  The most commonly reported side effects of IL-23 inhibitors, which are used to treat conditions like psoriasis, include: Upper Respiratory Tract Infections: These are the most frequently observed side effects, often presenting as mild infections such as the common cold. Headache: Another common side effect reported by patients using IL-23 inhibitors. Injection Site Reactions: Patients often experience mild to moderate reactions at the site of the injection, including redness, swelling, or pain. Fatigue: Some individuals report feeling unusually tired after using these medications. Gastrointestinal Issues: Nausea, diarrhea, and other gastrointestinal symptoms have been reported, though they tend to be mild.  IL-23 inhibitors are generally well-tolerated, but more serious adverse effects, though rare, can include the risk of serious infections and hypersensitivity reactions. Monitoring is essential, especially during long-term use.  **B**  Blauvelt, A., Papp, K. A., Griffiths, C. E. M., et al. (2017). Efficacy and safety of guselkumab, an interleukin-23–specific monoclonal antibody, compared with adalimumab for the treatment of moderate to severe psoriasis. *Lancet*, 390(10091), 228-238. doi:10.1016/S0140-6736(17)31216-3 |
| --- | --- |

**4. Describe the histological differences between eczema and psoriasis.**

**Answer (ChatGPT)**:

**Eczema (Atopic Dermatitis):**

- **Epidermal Changes:** Eczema is characterized by **spongiosis**, which is intercellular edema leading to a "spongy" appearance of the epidermis. This spongiosis can range from mild to severe and is typically accompanied by **acanthosis**, or thickening of the epidermis, particularly in chronic lesions. The epidermis in eczema also shows **parakeratosis** (retention of nuclei in the stratum corneum), and there can be **vesicle formation** in more severe cases.
- **Dermal Changes:** The dermis in eczema shows a **superficial perivascular infiltrate** of lymphocytes, with occasional eosinophils. There may also be **exocytosis** of lymphocytes into the epidermis.

**Psoriasis:**

- **Epidermal Changes:** Psoriasis is defined by **marked acanthosis** with regular elongation of the rete ridges. A key feature is **parakeratosis** along with the presence of **Munro's microabscesses** (collections of neutrophils in the stratum corneum). Unlike eczema, **spongiosis** is minimal or absent. Psoriasis also exhibits **hyperkeratosis** (thickening of the stratum corneum) and **hypogranulosis** (a reduction in the granular layer).
- **Dermal Changes:** In the dermis, there is a **dilated and tortuous capillary network** within the dermal papillae, leading to the characteristic Auspitz sign (pinpoint bleeding when scales are removed). The dermal infiltrate in psoriasis is primarily composed of T cells, dendritic cells, and neutrophils.

**Key Differences:**

- **Spongiosis** is prominent in eczema but minimal or absent in psoriasis.
- **Munro's microabscesses** and **regular acanthosis** are hallmarks of psoriasis but not seen in eczema.
- **Vesicle formation** is more characteristic of eczema, especially in acute lesions.

**Sources:**

1. **American Journal of Dermatopathology:** Link
2. **Journal of the American Academy of Dermatology:** [Link](https://www.jaad.org/)
3. **Dermatology: An Illustrated Colour Text** by Gawkrodger and Ardern-Jones.

**Answer (DermGPT)**: In eczema, histological examination typically reveals epidermal spongiosis, which is characterized by intercellular edema and separation of keratinocytes. There may also be a dense infiltration of inflammatory cells, including lymphocytes, eosinophils, and mast cells. Additionally, there can be hyperkeratosis, acanthosis (thickening of the epidermis), and parakeratosis (retention of nuclei in the stratum corneum) .On the other hand, psoriasis is characterized by epidermal hyperplasia, or psoriasiform hyperplasia, which is characterized by elongation of the rete ridges and thickening of the epidermis. The epidermis may exhibit regular acanthosis, with elongated and thin rete ridges. Another characteristic feature of psoriasis is the presence of Munro's microabscesses, which are collections of neutrophils in the stratum corneum. Additionally, there may be parakeratosis, hyperkeratosis, and a dense infiltration of inflammatory cells, predominantly T cells, in the dermis.

**Source**: Mansouri Y, Guttman-Yassky E. Immune Pathways in Atopic Dermatitis, and Definition of Biomarkers through Broad and Targeted Therapeutics. J Clin Med. 2015 Apr 29;4(5):858-73. doi: 10.3390/jcm4050858. PMID: 26239452; PMCID: PMC4470203

| **A**  Eczema (Atopic Dermatitis): Epidermal Changes: Eczema is characterized by spongiosis, which is intercellular edema leading to a "spongy" appearance of the epidermis. This spongiosis can range from mild to severe and is typically accompanied by acanthosis, or thickening of the epidermis, particularly in chronic lesions. The epidermis in eczema also shows parakeratosis (retention of nuclei in the stratum corneum), and there can be vesicle formation in more severe cases. Dermal Changes: The dermis in eczema shows a superficial perivascular infiltrate of lymphocytes, with occasional eosinophils. There may also be exocytosis of lymphocytes into the epidermis.  Psoriasis: Epidermal Changes: Psoriasis is defined by marked acanthosis with regular elongation of the rete ridges. A key feature is parakeratosis along with the presence of Munro's microabscesses (collections of neutrophils in the stratum corneum). Unlike eczema, spongiosis is minimal or absent. Psoriasis also exhibits hyperkeratosis (thickening of the stratum corneum) and hypogranulosis (a reduction in the granular layer). Dermal Changes: In the dermis, there is a dilated and tortuous capillary network within the dermal papillae, leading to the characteristic Auspitz sign (pinpoint bleeding when scales are removed). The dermal infiltrate in psoriasis is primarily composed of T cells, dendritic cells, and neutrophils.  Key Differences: Spongiosis is prominent in eczema but minimal or absent in psoriasis. Munro's microabscesses and regular acanthosis are hallmarks of psoriasis but not seen in eczema. Vesicle formation is more characteristic of eczema, especially in acute lesions.  **A**  American Journal of Dermatopathology  Journal of the American Academy of Dermatology  Dermatology: An Illustrated Colour Text by Gawkrodger and Ardern-Jones. | **B**  In eczema, histological examination typically reveals epidermal spongiosis, which is characterized by intercellular edema and separation of keratinocytes. There may also be a dense infiltration of inflammatory cells, including lymphocytes, eosinophils, and mast cells. Additionally, there can be hyperkeratosis, acanthosis (thickening of the epidermis), and parakeratosis (retention of nuclei in the stratum corneum) .On the other hand, psoriasis is characterized by epidermal hyperplasia, or psoriasiform hyperplasia, which is characterized by elongation of the rete ridges and thickening of the epidermis. The epidermis may exhibit regular acanthosis, with elongated and thin rete ridges. Another characteristic feature of psoriasis is the presence of Munro's microabscesses, which are collections of neutrophils in the stratum corneum. Additionally, there may be parakeratosis, hyperkeratosis, and a dense infiltration of inflammatory cells, predominantly T cells, in the dermis.  **B**  Mansouri Y, Guttman-Yassky E. Immune Pathways in Atopic Dermatitis, and Definition of Biomarkers through Broad and Targeted Therapeutics. J Clin Med. 2015 Apr 29;4(5):858-73. doi: 10.3390/jcm4050858. PMID: 26239452; PMCID: PMC4470203 |
| --- | --- |

**5. What is the etiology of Granuloma Annulare?**

**Answer (ChatGPT)**: Granuloma annulare (GA) is a chronic, inflammatory skin condition characterized by annular plaques or papules, typically occurring on the hands and feet. The exact etiology of granuloma annulare remains largely unknown, although several factors are thought to contribute to its development.

Research suggests that GA may result from a hypersensitivity reaction, possibly triggered by trauma, infections, or certain medications. There is also evidence pointing to an association with systemic conditions such as diabetes mellitus, thyroid disease, and, in some cases, malignancies. Immunologic factors, including altered T-cell responses and cytokine patterns, have been implicated in the pathogenesis of GA, indicating that the condition may involve a complex interaction between the immune system and external or internal triggers.

Despite ongoing research, the precise cause of granuloma annulare remains elusive, and more studies are needed to fully understand its etiology

**Source**: Dabski, K., & Winkelmann, R. K. (1989). Generalized granuloma annulare: Clinical and laboratory findings in 100 patients. *Journal of the American Academy of Dermatology*, 20(1), 39-47. doi:10.1016/S0190-9622(89)70006-1

Answer (DermGPT): The etiology of Granuloma Annulare (GA) remains uncertain. While the exact cause is unknown, several potential factors have been proposed. These include immune dysregulation, genetic predisposition, infections, and certain medications.

Source: Barbieri JS, Rodriguez O, Rosenbach M, Margolis D. Incidence and Prevalence of Granuloma Annulare in the United States. JAMA Dermatol. 2021 Jul 1;157(7):824-830. doi: 10.1001/jamadermatol.2021.1847. PMID: 34106215; PMCID: PMC8190699.

| **A**  The etiology of Granuloma Annulare (GA) remains uncertain. While the exact cause is unknown, several potential factors have been proposed. These include immune dysregulation, genetic predisposition, infections, and certain medications.  **A**  Barbieri JS, Rodriguez O, Rosenbach M, Margolis D. Incidence and Prevalence of Granuloma Annulare in the United States. JAMA Dermatol. 2021 Jul 1;157(7):824-830. doi: 10.1001/jamadermatol.2021.1847. PMID: 34106215; PMCID: PMC8190699. | **B**  Granuloma annulare (GA) is a chronic, inflammatory skin condition characterized by annular plaques or papules, typically occurring on the hands and feet. The exact etiology of granuloma annulare remains largely unknown, although several factors are thought to contribute to its development.  Research suggests that GA may result from a hypersensitivity reaction, possibly triggered by trauma, infections, or certain medications. There is also evidence pointing to an association with systemic conditions such as diabetes mellitus, thyroid disease, and, in some cases, malignancies. Immunologic factors, including altered T-cell responses and cytokine patterns, have been implicated in the pathogenesis of GA, indicating that the condition may involve a complex interaction between the immune system and external or internal triggers.  Despite ongoing research, the precise cause of granuloma annulare remains elusive, and more studies are needed to fully understand its etiology  **B**  Dabski, K., & Winkelmann, R. K. (1989). Generalized granuloma annulare: Clinical and laboratory findings in 100 patients. *Journal of the American Academy of Dermatology*, 20(1), 39-47. doi:10.1016/S0190-9622(89)70006-1 |
| --- | --- |

**6. What dermatoses should be included in the differential diagnosis of a papulosquamous reaction pattern?**

**Answer (ChatGPT)**: The differential diagnosis of a papulosquamous reaction pattern includes psoriasis, lichen planus, pityriasis rosea, seborrheic dermatitis, secondary syphilis, and tinea corporis.

**Source**: Usatine, R. P., & Tinitigan, M. (2011). Diagnosis and treatment of psoriasis. *American Family Physician*, 84(4), 380-387.

Answer (DermGPT): When considering a papulosquamous reaction pattern, several dermatoses should be included in the differential diagnosis. These include psoriasis, lichen planus, pityriasis rosea, seborrheic dermatitis, secondary syphilis, drug eruptions.

**Source**: Ernst M, Lundgren M, Evans MD, Miller D, Giubellino A. The mixed spongiotic and interface reaction pattern: A study of clinical and histopathologic findings. J Cutan Pathol. 2022 Dec;49(12):1051-1059. doi: 10.1111/cup.14306. Epub 2022 Aug 18. PMID: 36445270; PMCID: PMC9709294.

| **A**  The differential diagnosis of a papulosquamous reaction pattern includes psoriasis, lichen planus, pityriasis rosea, seborrheic dermatitis, secondary syphilis, and tinea corporis.  **A**  Usatine, R. P., & Tinitigan, M. (2011). Diagnosis and treatment of psoriasis. *American Family Physician*, 84(4), 380-387. | **B**  When considering a papulosquamous reaction pattern, several dermatoses should be included in the differential diagnosis. These include psoriasis, lichen planus, pityriasis rosea, seborrheic dermatitis, secondary syphilis, drug eruptions.  **B**  Ernst M, Lundgren M, Evans MD, Miller D, Giubellino A. The mixed spongiotic and interface reaction pattern: A study of clinical and histopathologic findings. J Cutan Pathol. 2022 Dec;49(12):1051-1059. doi: 10.1111/cup.14306. Epub 2022 Aug 18. PMID: 36445270; PMCID: PMC9709294. |
| --- | --- |

**7. Bullous Pemphigoid is caused by an autoantibody response directed against which antigens?**

**Answer (ChatGPT)**: Bullous Pemphigoid is caused by an autoantibody response directed against two specific antigens located in the hemidesmosomes, which are structures that help anchor the epidermis to the dermis:

**BP180 (also known as Type XVII Collagen or BPAG2)**: This is a transmembrane protein that spans the basal membrane and plays a crucial role in dermal-epidermal adhesion.

**BP230 (also known as BPAG1)**: This is an intracellular protein associated with the inner plaque of hemidesmosomes.

Autoantibodies targeting these antigens lead to the separation of the epidermis from the dermis, causing subepidermal blistering, which is characteristic of Bullous Pemphigoid.

**Source**: Di Zenzo, G., Dilling, A., Aho, S., et al. (2008). "Pemphigoid Diseases: Pathogenesis, Diagnosis, and Clinical Features." *Journal of Investigative Dermatology*, 128(1), 25-30.

**Answer (DermGPT):** Bullous Pemphigoid (BP) is caused by an autoantibody response directed against BP antigens 180 and 230 . These autoantibodies target proteins located at the dermoepidermal junction, leading to the detachment of the epidermis from the underlying dermis and the formation of blisters. The dominant epitope targeted by these autoantibodies is the NC16A domain of BP180 . The presence of these autoantibodies and their interaction with BP antigens play a crucial role in the pathogenesis of Bullous Pemphigoid.

**Source**: Bullous Pemphigoid Triggered by Liraglutide. Cutis. 2021 May;107(05):E9-E11 | doi:10.12788/cutis.0262. Mary-Katharine Collins, MD Sonal Choudhary, MD Jonhan Ho, MD Yuri L. Bunimovich, MD, PhD

**Second source**: Shen WC, Chiang HY, Chen PS, Lin YT, Kuo CC, Wu PY. Risk of All-Cause Mortality, Cardiovascular Disease Mortality, and Cancer Mortality in Patients With Bullous Pemphigoid. JAMA Dermatol. 2022 Feb 1;158(2):167-175. doi: 10.1001/jamadermatol.2021.5125. PMID: 34964804; PMCID: PMC8717210.

| **A**  Bullous Pemphigoid is caused by an autoantibody response directed against two specific antigens located in the hemidesmosomes, which are structures that help anchor the epidermis to the dermis: BP180 (also known as Type XVII Collagen or BPAG2): This is a transmembrane protein that spans the basal membrane and plays a crucial role in dermal-epidermal adhesion.  BP230 (also known as BPAG1): This is an intracellular protein associated with the inner plaque of hemidesmosomes.  Autoantibodies targeting these antigens lead to the separation of the epidermis from the dermis, causing subepidermal blistering, which is characteristic of Bullous Pemphigoid.  **A**  Di Zenzo, G., Dilling, A., Aho, S., et al. (2008). "Pemphigoid Diseases: Pathogenesis, Diagnosis, and Clinical Features." *Journal of Investigative Dermatology*, 128(1), 25-30. | **B**  Bullous Pemphigoid (BP) is caused by an autoantibody response directed against BP antigens 180 and 230 . These autoantibodies target proteins located at the dermoepidermal junction, leading to the detachment of the epidermis from the underlying dermis and the formation of blisters. The dominant epitope targeted by these autoantibodies is the NC16A domain of BP180 . The presence of these autoantibodies and their interaction with BP antigens play a crucial role in the pathogenesis of Bullous Pemphigoid.  **B**  Bullous Pemphigoid Triggered by Liraglutide. Cutis. 2021 May;107(05):E9-E11 \| doi:10.12788/cutis.0262. Mary-Katharine Collins, MD Sonal Choudhary, MD Jonhan Ho, MD Yuri L. Bunimovich, MD, PhD  And  Shen WC, Chiang HY, Chen PS, Lin YT, Kuo CC, Wu PY. Risk of All-Cause Mortality, Cardiovascular Disease Mortality, and Cancer Mortality in Patients With Bullous Pemphigoid. JAMA Dermatol. 2022 Feb 1;158(2):167-175. doi: 10.1001/jamadermatol.2021.5125. PMID: 34964804; PMCID: PMC8717210. |
| --- | --- |

**8. What are the guidelines for prescribing isotretinoin?**

**Answer (ChatGPT)**: The guidelines for prescribing isotretinoin include confirming the diagnosis of severe, recalcitrant nodular acne, obtaining informed consent, performing baseline lab tests, and adhering to the iPledge program, which requires regular monitoring and strict pregnancy prevention measures for female patients.

**Source**: Zaenglein, A. L., Pathy, A. L., Schlosser, B. J., et al. (2016). Guidelines of care for the management of acne vulgaris. *Journal of the American Academy of Dermatology*, 74(5), 945-973.e33. doi:10.1016/j.jaad.2015.12.037

**Answer (DermGPT)**:Isotretinoin is strongly recommended for severe acne, acne causing psychosocial burden or scarring, or acne failing standard treatment with oral or topical therapy: For patients with severe acne or those who have failed standard treatment, isotretinoin is recommended, For patients prescribed isotretinoin, monitoring of liver function tests (LFTs) and lipids should be considered, but complete blood count (CBC) monitoring is not needed in healthy patients 1, Traditional daily dosing of isotretinoin is conditionally recommended over intermittent dosing for patients with severe acne, The choice between standard isotretinoin and lidose-isotretinoin can be made based on individual patient factors.

**Source**: Guidelines of care for the management of acne vulgaris. J Am Acad Dermatol. 2024;1.e1-1.e2. doi:10.1016/j.jaad.2023.12.017

| **A**  Isotretinoin is strongly recommended for severe acne, acne causing psychosocial burden or scarring, or acne failing standard treatment with oral or topical therapy: For patients with severe acne or those who have failed standard treatment, isotretinoin is recommended, For patients prescribed isotretinoin, monitoring of liver function tests (LFTs) and lipids should be considered, but complete blood count (CBC) monitoring is not needed in healthy patients 1, Traditional daily dosing of isotretinoin is conditionally recommended over intermittent dosing for patients with severe acne, The choice between standard isotretinoin and lidose-isotretinoin can be made based on individual patient factors.  **A**  Guidelines of care for the management of acne vulgaris. J Am Acad Dermatol. 2024;1.e1-1.e2. doi:10.1016/j.jaad.2023.12.017 | **B**  The guidelines for prescribing isotretinoin include confirming the diagnosis of severe, recalcitrant nodular acne, obtaining informed consent, performing baseline lab tests, and adhering to the iPledge program, which requires regular monitoring and strict pregnancy prevention measures for female patients.  **B**  Zaenglein, A. L., Pathy, A. L., Schlosser, B. J., et al. (2016). Guidelines of care for the management of acne vulgaris. *Journal of the American Academy of Dermatology*, 74(5), 945-973.e33. doi:10.1016/j.jaad.2015.12.037 |
| --- | --- |

**9. What is the classic clinical appearance of calciphylaxis?**

**Answer (ChatGPT)**:

1. **Painful Skin Lesions**: Calciphylaxis typically presents with extremely painful, violaceous (purple) to black, retiform (net-like) purpuric plaques or nodules. These lesions are often tender and rapidly progress to necrosis.
2. **Ulceration and Necrosis**: The lesions frequently ulcerate, leading to the development of eschar (dead tissue), which may be black or brown. The surrounding skin may show signs of erythema (redness) and induration (hardening).
3. **Distribution**: Lesions are most commonly found on areas with high adipose tissue, such as the thighs, buttocks, and abdomen, but they can also occur on other parts of the body, including the breasts and lower legs.
4. **Gangrene**: In severe cases, calciphylaxis can lead to gangrene, which may result in significant tissue loss and systemic infection.

These lesions result from vascular calcification and subsequent ischemia, primarily affecting patients with end-stage renal disease.

**Source**: Weenig, R. H., Sewell, L. D., Davis, M. D. P., McCarthy, J. T., & Pittelkow, M. R. (2007). "Calciphylaxis: Natural history, risk factor analysis, and outcome." *Journal of the American Academy of Dermatology*, 56(4), 569-579.

**Answer (DermGPT):** The classic clinical appearance of calciphylaxis is characterized by indurated plaques with overlying retiform purpura and central necrosis. These plaques typically evolve into stellate ulcerations over the abdomen, thighs, and buttocks. It is important to note that the clinical presentation of calciphylaxis can vary, and the location of ulcerations may differ depending on the subtype of calciphylaxis. For example, warfarin-associated calciphylaxis tends to ulcerate below the knee, while ulcerations of classic calciphylaxis are usually proximal and over fat-bearing regions.

Source: "Warfarin-Associated Nonuremic Calciphylaxis" - Wesley Yung-Hsu Yu, MD, et al.

| **A**  The classic clinical appearance of calciphylaxis is characterized by indurated plaques with overlying retiform purpura and central necrosis. These plaques typically evolve into stellate ulcerations over the abdomen, thighs, and buttocks. It is important to note that the clinical presentation of calciphylaxis can vary, and the location of ulcerations may differ depending on the subtype of calciphylaxis. For example, warfarin-associated calciphylaxis tends to ulcerate below the knee, while ulcerations of classic calciphylaxis are usually proximal and over fat-bearing regions.  **A**  "Warfarin-Associated Nonuremic Calciphylaxis" - Wesley Yung-Hsu Yu, MD, et al. | **B**  Painful Skin Lesions: Calciphylaxis typically presents with extremely painful, violaceous (purple) to black, retiform (net-like) purpuric plaques or nodules. These lesions are often tender and rapidly progress to necrosis. Ulceration and Necrosis: The lesions frequently ulcerate, leading to the development of eschar (dead tissue), which may be black or brown. The surrounding skin may show signs of erythema (redness) and induration (hardening). Distribution: Lesions are most commonly found on areas with high adipose tissue, such as the thighs, buttocks, and abdomen, but they can also occur on other parts of the body, including the breasts and lower legs. Gangrene: In severe cases, calciphylaxis can lead to gangrene, which may result in significant tissue loss and systemic infection. These lesions result from vascular calcification and subsequent ischemia, primarily affecting patients with end-stage renal disease.  **B**  Weenig, R. H., Sewell, L. D., Davis, M. D. P., McCarthy, J. T., & Pittelkow, M. R. (2007). "Calciphylaxis: Natural history, risk factor analysis, and outcome." *Journal of the American Academy of Dermatology*, 56(4), 569-579. |
| --- | --- |

**10. How do you rate the severity of Hidradenitis Suppurativa?**

**Answer (ChatGPT)**: The severity of Hidradenitis Suppurativa (HS) is commonly rated using the **Hurley staging system**, which categorizes the disease into three stages based on the extent and type of lesions:

1. **Hurley Stage I**:
   - **Mild**: Characterized by the presence of single or multiple abscesses without sinus tracts (tunnels under the skin) or scarring.
   - Lesions are isolated and localized.
2. **Hurley Stage II**:
   - **Moderate**: Involves recurrent abscesses with the formation of sinus tracts and scarring.
   - The lesions are more widely separated, with multiple areas of involvement.
3. **Hurley Stage III**:
   - **Severe**: This stage is marked by diffuse involvement or multiple interconnected sinus tracts and abscesses across an entire area of the body.
   - Extensive scarring and chronic lesions are present, often leading to significant morbidity.

The Hurley staging system is widely used in clinical practice to assess the severity of HS and to guide treatment decisions.

**Source**: Hurley, H. J. (1989). *Diseases of the Skin* (9th ed.). Saunders.

Answer (DermGPT): One such tool is the Hidradenitis Suppurativa Area and Severity Index Revised (HASI-R), which is a clinician-reported outcome measure 1 . The HASI-R incorporates signs of inflammation, such as erythema, thickness, drainage, and tenderness, as well as the extent of involvement of body surface area (BSA). It provides a comprehensive assessment of HS severity by considering both the physical signs and the BSA involved. Another tool commonly used is the International HS Severity Score System (IHS4), which is a dynamic scoring system that assesses HS severity based on the number of inflammatory nodules, abscesses, and draining tunnels, each with predetermined weights. The IHS4 takes into account the severity of the physical signs of HS and provides a quantitative measure of disease severity.

Source(s): Hidradenitis Suppurativa Area and Severity Index Revised (HASI-R): psychometric property assessment **and** Development and validation of the International Hidradenitis Suppurativa Severity Score System (IHS4), a novel dynamic scoring system to assess HS severity

| **A**  The severity of Hidradenitis Suppurativa (HS) is commonly rated using the Hurley staging system, which categorizes the disease into three stages based on the extent and type of lesions:  Hurley Stage I:  Mild: Characterized by the presence of single or multiple abscesses without sinus tracts (tunnels under the skin) or scarring.  Lesions are isolated and localized.  Hurley Stage II:  Moderate: Involves recurrent abscesses with the formation of sinus tracts and scarring.  The lesions are more widely separated, with multiple areas of involvement.  Hurley Stage III:  Severe: This stage is marked by diffuse involvement or multiple interconnected sinus tracts and abscesses across an entire area of the body.  Extensive scarring and chronic lesions are present, often leading to significant morbidity.  **A**  Hurley, H. J. (1989). *Diseases of the Skin* (9th ed.). Saunders. | **B**  One such tool is the Hidradenitis Suppurativa Area and Severity Index Revised (HASI-R), which is a clinician-reported outcome measure 1 . The HASI-R incorporates signs of inflammation, such as erythema, thickness, drainage, and tenderness, as well as the extent of involvement of body surface area (BSA). It provides a comprehensive assessment of HS severity by considering both the physical signs and the BSA involved. Another tool commonly used is the International HS Severity Score System (IHS4), which is a dynamic scoring system that assesses HS severity based on the number of inflammatory nodules, abscesses, and draining tunnels, each with predetermined weights. The IHS4 takes into account the severity of the physical signs of HS and provides a quantitative measure of disease severity.  **B**  Hidradenitis Suppurativa Area and Severity Index Revised (HASI-R): psychometric property assessment **and** Development and validation of the International Hidradenitis Suppurativa Severity Score System (IHS4), a novel dynamic scoring system to assess HS severity |
| --- | --- |

**11. What margin should be used for lentigo maligna (or severely dysplastic nevus)?**

**Answer (ChatGPT)**: For lentigo maligna, the recommended surgical margin is typically **5-10 mm**. For a severely dysplastic nevus, the margin is generally **2-5 mm** depending on the degree of atypia and the clinical context.

**Source**: Bichakjian, C. K., Halpern, A. C., Johnson, T. M., et al. (2011). Guidelines of care for the management of primary cutaneous melanoma. *Journal of the American Academy of Dermatology*, 65(5), 1032-1047. doi:10.1016/j.jaad.2011.04.031

**Answer (DermGPT):** The recommended margin for lentigo maligna or severely dysplastic nevus excision is typically 5 mm. This margin is used to ensure complete removal of the lesion and reduce the risk of recurrence.

**Source:** Staged excision for lentigo maligna and lentigo maligna melanoma: a retrospective analysis of 117 cases - Zalaudek I, et al.

| **A**  For lentigo maligna, the recommended surgical margin is typically 5-10 mm. For a severely dysplastic nevus, the margin is generally 2-5 mm depending on the degree of atypia and the clinical context.  **A**  Bichakjian, C. K., Halpern, A. C., Johnson, T. M., et al. (2011). Guidelines of care for the management of primary cutaneous melanoma. *Journal of the American Academy of Dermatology*, 65(5), 1032-1047. doi:10.1016/j.jaad.2011.04.031 | **B**  The recommended margin for lentigo maligna or severely dysplastic nevus excision is typically 5 mm. This margin is used to ensure complete removal of the lesion and reduce the risk of recurrence.  **B**  Staged excision for lentigo maligna and lentigo maligna melanoma: a retrospective analysis of 117 cases - Zalaudek I, et al. |
| --- | --- |

12. **Which is the best biologic for psoriasis?**

**Answer (ChatGPT):** The choice of the "best" biologic for psoriasis depends on various factors, including the severity of the disease, patient characteristics, comorbid conditions, and the specific clinical goals (e.g., rapid clearance, long-term maintenance, safety profile). However, IL-17 inhibitors and IL-23 inhibitors are currently considered among the most effective biologics for treating moderate to severe psoriasis.

1. IL-17 Inhibitors:
   - Secukinumab (Cosentyx) and Ixekizumab (Taltz) are IL-17 inhibitors that have demonstrated high efficacy in clearing psoriatic plaques. They work by targeting IL-17A, a cytokine involved in the inflammatory process of psoriasis. Both drugs have shown rapid and sustained skin clearance in clinical trials.
2. IL-23 Inhibitors:
   - Guselkumab (Tremfya), Risankizumab (Skyrizi), and Tildrakizumab (Ilumya) are IL-23 inhibitors that target the p19 subunit of IL-23, a cytokine that plays a central role in psoriasis pathogenesis. These biologics have shown excellent efficacy and durability, with a favorable safety profile.

Studies have shown that these newer biologics, particularly IL-17 and IL-23 inhibitors, may offer superior skin clearance rates and long-term control compared to older biologics like TNF-alpha inhibitors (e.g., adalimumab, infliximab).

**Source**: Armstrong, A. W., et al. (2020). "Efficacy and safety of biologics for moderate-to-severe plaque psoriasis: A network meta-analysis." *Dermatology and Therapy*, 10(2), 117-137.

**Answer (DermGPT)**: A systematic review published in the journal "Biologics: Targets and Therapy" provides a comprehensive assessment of the efficacy and safety of FDA-approved biologic agents for psoriasis. The review discusses the clinically useful facts about each FDA-approved biologic agent, including their unique merits and demerits, to help healthcare providers select the most suitable option for individual patients.

**Source**: "Biologic Treatments of Psoriasis: An Update for the Clinician" - Brownstone ND, et al.

| **A**  The choice of the "best" biologic for psoriasis depends on various factors, including the severity of the disease, patient characteristics, comorbid conditions, and the specific clinical goals (e.g., rapid clearance, long-term maintenance, safety profile). However, IL-17 inhibitors and IL-23 inhibitors are currently considered among the most effective biologics for treating moderate to severe psoriasis. IL-17 Inhibitors: Secukinumab (Cosentyx) and Ixekizumab (Taltz) are IL-17 inhibitors that have demonstrated high efficacy in clearing psoriatic plaques. They work by targeting IL-17A, a cytokine involved in the inflammatory process of psoriasis. Both drugs have shown rapid and sustained skin clearance in clinical trials. IL-23 Inhibitors: Guselkumab (Tremfya), Risankizumab (Skyrizi), and Tildrakizumab (Ilumya) are IL-23 inhibitors that target the p19 subunit of IL-23, a cytokine that plays a central role in psoriasis pathogenesis. These biologics have shown excellent efficacy and durability, with a favorable safety profile.  Studies have shown that these newer biologics, particularly IL-17 and IL-23 inhibitors, may offer superior skin clearance rates and long-term control compared to older biologics like TNF-alpha inhibitors (e.g., adalimumab, infliximab).  **A**  Armstrong, A. W., et al. (2020). "Efficacy and safety of biologics for moderate-to-severe plaque psoriasis: A network meta-analysis." *Dermatology and Therapy*, 10(2), 117-137. | **B**  A systematic review published in the journal "Biologics: Targets and Therapy" provides a comprehensive assessment of the efficacy and safety of FDA-approved biologic agents for psoriasis. The review discusses the clinically useful facts about each FDA-approved biologic agent, including their unique merits and demerits, to help healthcare providers select the most suitable option for individual patients.  **B**  "Biologic Treatments of Psoriasis: An Update for the Clinician" - Brownstone ND, et al. |
| --- | --- |

**13. What is the skin cancer screening recommendation for the general population?**

Answer (ChatGPT): For the general population, routine skin cancer screening by a healthcare professional is not universally recommended. However, individuals are encouraged to perform regular skin self-examinations and report any suspicious lesions to a healthcare provider. Those at high risk, such as individuals with a history of skin cancer or extensive sun exposure, may benefit from regular screenings by a dermatologist.

Source: US Preventive Services Task Force. (2016). Screening for Skin Cancer: US Preventive Services Task Force Recommendation Statement. JAMA, 316(4), 429-435. doi:10.1001/jama.2016.8465

Answer (DermGPT) : The skin cancer screening recommendation for the general population is not to conduct routine population-based skin cancer screening. The United States Preventive Services Task Force (USPSTF) does not recommend routine screening for skin cancer in individuals without a history of premalignant skin lesions, skin cancer, suspicious skin lesions, or increased risk of skin cancer based on abnormal mole phenotype or family history of melanoma. The USPSTF has determined that the current evidence is insufficient to assess the balance of benefits and harms of routine skin cancer screening.

Sources: Screening for skin cancer: U.S. Preventive Services Task Force recommendation statement" - USPSTF ,Screening for Skin Cancer: An Update of the Evidence for the US Preventive Services Task Force", Screening for skin cancer in adults: an updated systematic evidence review for the U.S. Preventive Services Task Force

| **A**  The skin cancer screening recommendation for the general population is not to conduct routine population-based skin cancer screening. The United States Preventive Services Task Force (USPSTF) does not recommend routine screening for skin cancer in individuals without a history of premalignant skin lesions, skin cancer, suspicious skin lesions, or increased risk of skin cancer based on abnormal mole phenotype or family history of melanoma. The USPSTF has determined that the current evidence is insufficient to assess the balance of benefits and harms of routine skin cancer screening.  **A**  Screening for skin cancer: U.S. Preventive Services Task Force recommendation statement" - USPSTF ,Screening for Skin Cancer: An Update of the Evidence for the US Preventive Services Task Force", Screening for skin cancer in adults: an updated systematic evidence review for the U.S. Preventive Services Task Force | **B**  For the general population, routine skin cancer screening by a healthcare professional is not universally recommended. However, individuals are encouraged to perform regular skin self-examinations and report any suspicious lesions to a healthcare provider. Those at high risk, such as individuals with a history of skin cancer or extensive sun exposure, may benefit from regular screenings by a dermatologist.  **B**  US Preventive Services Task Force. (2016). Screening for Skin Cancer: US Preventive Services Task Force Recommendation Statement. JAMA, 316(4), 429-435. doi:10.1001/jama.2016.8465 |
| --- | --- |

14. **What dermatoses should be included in the differential diagnosis of an eczematous reaction pattern?**

**Answer (ChatGPT)**: The differential diagnosis for an eczematous reaction pattern includes atopic dermatitis, irritant contact dermatitis, allergic contact dermatitis, seborrheic dermatitis, nummular dermatitis, lichen simplex chronicus, stasis dermatitis, tinea corporis, psoriasis, drug reactions, scabies.

**Source**:James, W. D., Elston, D. M., Treat, J. R., Rosenbach, M. A., & Neuhaus, I. M. (2020). *Andrews' Diseases of the Skin: Clinical Dermatology* (13th ed.). Elsevier.

**Answer (DermGPT)**: When considering the differential diagnosis of an eczematous reaction pattern, several dermatoses should be included. These may include: Atopic dermatitis, Contact dermatitis, Seborrheic dermatitis, Nummular dermatitis, Dyshidrotic eczema, Psoriasis.

**Source**: Robinson CA, Love LW, Saleh HM, et al. Nummular Dermatitis. [Updated 2024 Mar 1]. In: StatPearls [Internet]. Treasure Island (FL): StatPearls Publishing; 2024 Jan-. Available from: <https://www.ncbi.nlm.nih.gov/books/NBK565878/>

**Second source:** Litchman G, Nair PA, Atwater AR, et al. Contact Dermatitis. [Updated 2023 Sep 4]. In: StatPearls [Internet]. Treasure Island (FL): StatPearls Publishing; 2024 Jan-. Available from: <https://www.ncbi.nlm.nih.gov/books/NBK459230/>

| **A**  The differential diagnosis for an eczematous reaction pattern includes atopic dermatitis, irritant contact dermatitis, allergic contact dermatitis, seborrheic dermatitis, nummular dermatitis, lichen simplex chronicus, stasis dermatitis, tinea corporis, psoriasis, drug reactions, scabies.  **A**  James, W. D., Elston, D. M., Treat, J. R., Rosenbach, M. A., & Neuhaus, I. M. (2020). *Andrews' Diseases of the Skin: Clinical Dermatology* (13th ed.). Elsevier. | **B**  When considering the differential diagnosis of an eczematous reaction pattern, several dermatoses should be included. These may include: Atopic dermatitis, Contact dermatitis, Seborrheic dermatitis, Nummular dermatitis, Dyshidrotic eczema, Psoriasis.  **B**  Robinson CA, Love LW, Saleh HM, et al. Nummular Dermatitis. [Updated 2024 Mar 1]. In: StatPearls [Internet]. Treasure Island (FL): StatPearls Publishing; 2024 Jan-. Available from: <https://www.ncbi.nlm.nih.gov/books/NBK565878/> AND  Litchman G, Nair PA, Atwater AR, et al. Contact Dermatitis. [Updated 2023 Sep 4]. In: StatPearls [Internet]. Treasure Island (FL): StatPearls Publishing; 2024 Jan-. Available from: <https://www.ncbi.nlm.nih.gov/books/NBK459230/> |
| --- | --- |

15. **What medications are used to treat hormonally exacerbated acne?**

**Answer** (ChatGPT)

**Oral Contraceptives**:

- Combined oral contraceptives (COCs) containing both estrogen and progestin are commonly used. They help reduce androgen levels and decrease sebum production. Examples include:
  - **Ethinyl estradiol with norgestimate (Ortho Tri-Cyclen)**
  - **Ethinyl estradiol with drospirenone (Yaz, Yasmin)**
  - **Ethinyl estradiol with norethindrone (Estrostep)**

**Spironolactone**:

- A potassium-sparing diuretic that also has anti-androgenic effects. It works by blocking androgen receptors and inhibiting androgen production, which helps reduce sebum production and acne formation.

**Anti-androgens**:

- While less commonly used, **flutamide** and **cyproterone acetate** are examples of anti-androgens that may be used in cases where acne is particularly resistant to other treatments. However, these are typically not first-line treatments due to potential side effects.

**Topical Agents**:

- While not specifically targeting hormones, topical retinoids, benzoyl peroxide, and antibiotics are often used in combination with hormonal treatments to address acne more comprehensively.

**Source**: Zaenglein, A. L., Pathy, A. L., Schlosser, B. J., et al. (2016). "Guidelines of care for the management of acne vulgaris." *Journal of the American Academy of Dermatology*, 74(5), 945-973.

Answer (DermGPT): The medications commonly used to treat hormonally exacerbated acne include oral contraceptives, spironolactone, and anti-androgen medications.

Source: Guidelines of care for the management of acne vulgaris. J Am Acad Dermatol. 2024;1.e1-1.e2. doi:10.1016/j.jaad.2023.12.017.

| **A**  The medications commonly used to treat hormonally exacerbated acne include oral contraceptives, spironolactone, and anti-androgen medications.  **A**  Guidelines of care for the management of acne vulgaris. J Am Acad Dermatol. 2024;1.e1-1.e2. doi:10.1016/j.jaad.2023.12.017. | **B**  Combined oral contraceptives (COCs) containing both estrogen and progestin are commonly used. They help reduce androgen levels and decrease sebum production. Examples include:  Ethinyl estradiol with norgestimate (Ortho Tri-Cyclen), Ethinyl estradiol with drospirenone (Yaz, Yasmin), Ethinyl estradiol with norethindrone (Estrostep)  Spironolactone: A potassium-sparing diuretic that also has anti-androgenic effects. It works by blocking androgen receptors and inhibiting androgen production, which helps reduce sebum production and acne formation.  Anti-androgens: While less commonly used, flutamide and cyproterone acetate are examples of anti-androgens that may be used in cases where acne is particularly resistant to other treatments. However, these are typically not first-line treatments due to potential side effects. Topical Agents:  While not specifically targeting hormones, topical retinoids, benzoyl peroxide, and antibiotics are often used in combination with hormonal treatments to address acne more comprehensively.  **B**  Zaenglein, A. L., Pathy, A. L., Schlosser, B. J., et al. (2016). "Guidelines of care for the management of acne vulgaris." *Journal of the American Academy of Dermatology*, 74(5), 945-973. |
| --- | --- |

DermGPT omitted questions and answers below that were not a part of the survey:

**10. What is the life expectancy of a patient with newly diagnosed stage 4B CTCL?**

**Answer (ChatGPT)**: The life expectancy for a patient with newly diagnosed stage 4B Cutaneous T-cell Lymphoma (CTCL) varies significantly, but it is generally poor. The median survival is often less than 2 years, depending on factors like the patient’s overall health, response to treatment, and specific subtype of CTCL.

**Source**: Scarisbrick, J. J., Quaglino, P., Prince, H. M., et al. (2015). The PROCLIPI international registry of early-stage mycosis fungoides identifies substantial diagnostic delay in most patients. *British Journal of Dermatology*, 173(5), 1298-1303. doi:10.1111/bjd.13998.

Answer (DermGPT): The retrieved documents do not provide specific information about the life expectancy of a patient with newly diagnosed stage 4B CTCL. It is important to consult with a dermatologist or medical professional who can provide accurate and up-to-date information on prognosis and life expectancy for individual cases of stage 4B CTCL. They can consider various factors such as the patient's overall health, response to treatment, and specific characteristics of the disease to provide a more personalized prognosis. The requested information is not available in the retrieved data. Please try another query or topic.

Source: n/a

24. What are the recommended surgical margins for excising a Melanoma with a Breslow depth of 0.8 mm?

Answer (ChatGPT): The recommended surgical margins for excising a melanoma with a Breslow depth of 0.8 mm are generally 1 cm.

Source: NCCN Clinical Practice Guidelines in Oncology (NCCN Guidelines®) for Melanoma: Cutaneous (Version 3.2024). National Comprehensive Cancer Network.

Answer (DermGPT): The recommended surgical margins for excising a Melanoma with a Breslow depth of 0.8 mm are not mentioned in the retrieved documents. Therefore, I cannot provide a specific answer with a scientific journal or authoritative source. It is recommended to consult dermatology guidelines or consult with a dermatologist for the most up-to-date and accurate information regarding surgical margins for excising melanoma with a specific Breslow depth.

Source: n/a

25. Is mohs micrographic surgery recommended for an invasive 0.6mm melanoma?

Answer (ChatGPT): Mohs micrographic surgery is generally not recommended for the treatment of an invasive melanoma, including one with a Breslow depth of 0.6 mm. Mohs surgery is typically reserved for non-melanoma skin cancers, such as basal cell carcinoma and squamous cell carcinoma, where precise margin control is crucial for preserving tissue, particularly in cosmetically sensitive areas.

For invasive melanoma, including one with a Breslow depth of 0.6 mm, the standard treatment is wide local excision with appropriate surgical margins. This approach ensures complete removal of the melanoma with a margin of healthy tissue, typically 1 cm for melanomas with a Breslow depth of less than 1 mm. Mohs surgery is not commonly used because melanoma cells can spread beyond the visible tumor, making it challenging to accurately assess margins during the procedure.

Source: Bichakjian, C.K., Halpern, A.C., Johnson, T.M., et al. (2011). "Guidelines of care for the management of primary cutaneous melanoma." Journal of the American Academy of Dermatology, 65(5), 1032-1047. DOI: 10.1016/j.jaad.2011.04.031

Answer (DermGPT): Mohs micrographic surgery (MMS) is a surgical technique commonly used for the treatment of skin cancers, including melanoma. However, the specific recommendation for MMS in the case of an invasive 0.6mm melanoma is not mentioned in the retrieved documents. To obtain the most accurate and up-to-date information, it is recommended to consult dermatology guidelines or consult with a dermatologist who can provide personalized recommendations based on the individual patient's characteristics and the specific features of the melanoma.

Source: n/a

*Initial Questions:*

1. What is seborrheic keratosis?
2. What is the best treatment for mild psoriasis?
3. What is the difference between an papule and a macule?
4. Name the layers of the skin from superficial to deep
5. How does sun exposure increase one’s risk for skin cancer?
6. What are the most reported side effects of IL-23 inhibitors?
7. Describe the histological differences between eczema and psoriasis.
8. What is the life expectancy of a patient with newly diagnosed stage 4B CTCL?
9. What is the etiology of Granuloma Annulare?
10. Can I use corticosteroids to treat SJS/TEN?
11. Bullous Pemphigoid is caused by an autoantibody response directed against which antigens?
12. What dermatoses should be included in the differential diagnosis of a papulosquamous reaction pattern?
13. What dermatoses should be included in the differential diagnosis of an eczematous reaction pattern?
14. What are the recommended surgical margins for excising a Melanoma with a Breslow depth of 0.8 mm?
15. What are the recommended surgical margins for excising a cutaneous squamous cell carcinoma?
16. What are the guidelines for prescribing isotretinoin?
17. What is the classic clinical appearance of calciphylaxis?
18. What medications are used to treat hormonally exacerbated acne?
19. How do you rate the severity of Hidradenitis Suppurativa?
20. How do you rate the severity of DRESS?
21. What gene is mutated in CHILD syndrome?
22. Is mohs micrographic surgery recommended for an invasive 0.6mm melanoma?
23. What is the skin cancer screening recommendation for the general population?
24. What margin should be used for lentigo maligna (or severely dysplastic nevus)?
25. Which is the best biologic for psoriasis?
