# Supplementary material for: Evaluating Artificial Intelligence Models in Dermatology: Comparative Analysis
Source: JMIR Dermatol. 2025 Dec 4;8:e74040. doi: 10.2196/74040 (PMC12677980; doi:10.2196/74040)
Supplement: Multimedia Appendix 7 [file derma-v8-e74040-s007.docx]

***Comments from the ChatGPT vs. DermGPT survey:***

1. I answered these as "better" answer for the physician/provider, but I don't think that necessarily holds true as the "better" answer when the patient looks up the question on their own.
2. Great idea for a study. I'd love to hear the preliminary results so I can start to apply the conclusions to my practice
